# Single‐Photon Emission From TMDCs through Strong Coupling to EQ Plasmon Modes of an Au Metasurface

**DOI:** 10.1002/nap2.70147

**Published:** 2026-05-29

**Authors:** Andergachew Mekonnen Berhe, Haroldo T. Hattori, Andrey E. Miroshnichenko

**Affiliations:** ^1^ School of Engineering and Technology University of New South Wales at Canberra Canberra Australian Capital Territory Australia

**Keywords:** antibunching, coupling strength, electric quadrupole, photon blockade, single photon purity

## Abstract

In this study, we theoretically demonstrate that a strongly coupled hybrid platform consisting of a compact gold plasmonic metamaterial (metasurface) and a monolayer of transition‐metal dichalcogenides (TMDCs) enables scalable single‐photon emission (SPE) at room temperature. The Au metasurface is designed to support electric‐quadrupole (EQ) resonance that efficiently couples to excitons in tungsten diselenide (${\text{WSe}}_{2}$), molybdenum disulfide (${\text{MoS}}_{2}$), and molybdenum diselenide (${\text{MoSe}}_{2}$). The planar metasurface geometry enables in‐plane field overlap with the TMDC exciton, yielding a pronounced coupling strength with a vacuum Rabi splitting exceeding 100 meV. To connect this enhanced coupling to our investigation of SPE purity, we first realized conventional photon blockade (CPB) in an Au–${\text{WSe}}_{2}$ hybrid under weak coherent driving, achieving $g^{\left(2\right)}\left(0\right)=4\times 10^{-4}$. We then demonstrated unconventional photon blockade (UnPB) in Au–${\text{WSe}}_{2}$, Au–${\text{MoS}}_{2}$, and Au–${\text{MoSe}}_{2}$ using a two‐tone coherent drive to induce destructive interference between two‐photon excitation pathways, resulting in very high purity. Additionally, pure dephasing of the emitters up to $\Gamma_{\phi }=40$ meV was considered, and antibunching was preserved. These results establish a scalable route to room‐temperature, high‐purity SPE for quantum communication and integrated quantum photonics.

## Graphical abstract

Compact, deterministic solid‐state single‐photon sources that operate at room temperature and integrate with planar nanophotonics are essential for the development of scalable quantum photonic technologies. This work presents a TMDC monolayer strongly coupled to a gold (Au) metasurface featuring an electric quadrupole mode, enabling high‐purity single‐photon emission through photon blockade by suppressing two‐photon occupation.

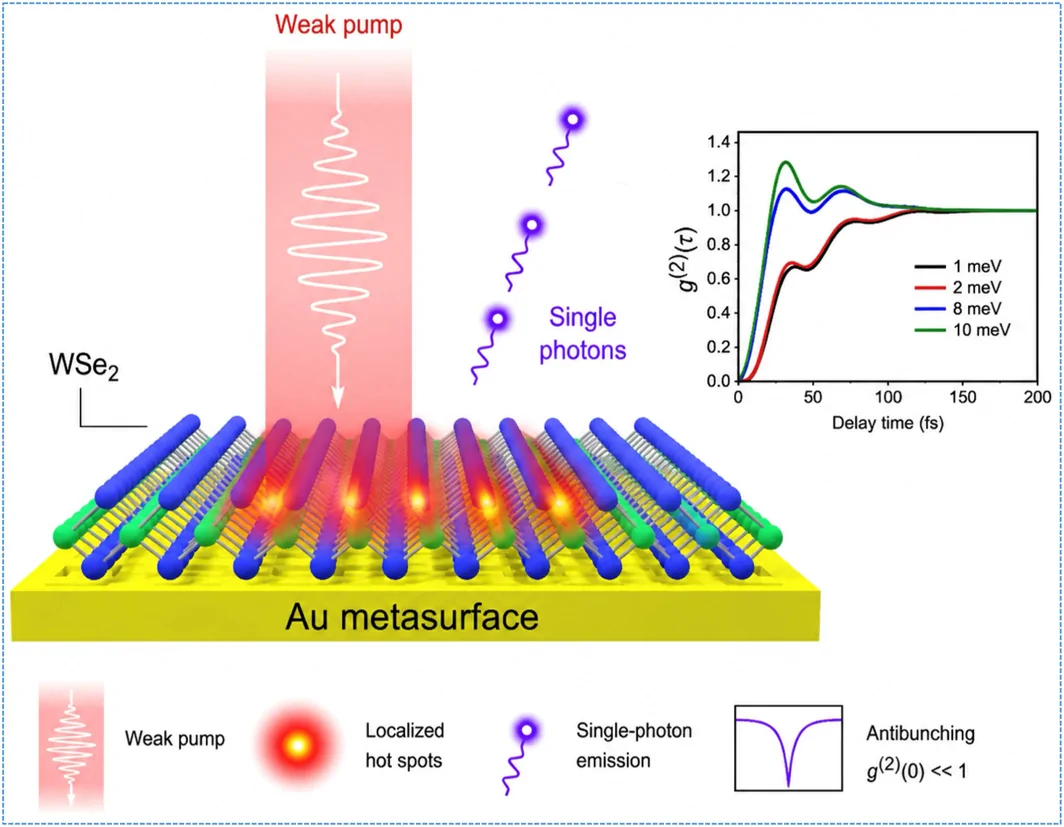

## 1 Introduction

Quantum nanophotonics provides a versatile platform for confining and coherently controlling individual quanta of light and matter at deeply sub‐wavelength scales, enabling deterministic single‐photon generation and on‐chip quantum functionality [1, 2]. In this context, single photons become a universal resource for encoding qubits across quantum communication and photonic quantum computing [3, 4, 5]. In quantum computing [6], photonic qubits support superposition and parallel processing, whereas in quantum communication [7], deterministic single‐photon sources (SPSs) underpin quantum key distribution and related cryptographic protocols with provable security. Subsequently, over the past decade, solid‐state platforms ranging from defect centers in diamond to semiconductor quantum dots (QDs), hexagonal boron nitride (hBN) defect emitters, and colloidal/perovskite QDs have been pursued as SPSs [8]. However, hBN defect emitters still face challenges associated with spectral diffusion, blinking, emitter‐to‐emitter variability, and deterministic placement of optically active defects, which can limit reproducibility [9]. Additionally, hBN defect emitters exhibit lower oscillator strengths compared with TMDCs, resulting in weaker light–matter coupling and lower achievable Rabi splittings in cavity‐coupled geometries [10]. Additionally, III–V epitaxial QDs generally require cryogenic operation and are sensitive to charge noise and phonon sidebands, forcing trade‐offs between brightness and indistinguishability unless complex cavity architectures are employed [11]. Besides, although these QDs have been integrated with optical metasurfaces to control emission directionality, spin state, and photon out‐coupling [12], they still rely on epitaxially embedded emitters and post‐growth nanophotonic patterning. In contrast, TMDC monolayers offer an atomically thin van der Waals emitter platform that can be dry‐transferred onto prefabricated photonic or plasmonic metasurface cavities, enabling flexible heterogeneous integration and strain‐defined localization. Moreover, colloidal perovskite QDs also suffer from limited photostability and environmental robustness, as well as concerns about material toxicity, despite recent advances in surface passivation [13]. Additionally, despite notable progress in TMDC‐based SPE, the operating temperature remains limited to below 150 K [14]. Thus, existing quantum light sources still face limited integrability, restricted spectral and spatial tunability, and inefficiencies in achieving deterministic on‐demand single‐photon generation [15].

To develop scalable, integrable SP emitters and operate them at room temperature, they should exhibit high emission efficiency and purity [16]. The purity of these photons can be measured through the second‐order correlation function at zero delay, $g^{\left(2\right)}\left(0\right)$, with an ideal value of $g^{\left(2\right)}\left(0\right)=0$, corresponding to unity purity defined as $P=1-g^{\left(2\right)}\left(0\right)$ [17]. As a result, one promising route to single‐photon generation with high brightness and purity is through cavity quantum electrodynamics (cQED) [3]. For instance, He et al. demonstrated the possibility of SPE from a bare ${\text{WSe}}_{2}$ monolayer under cryogenic conditions, achieving $g^{\left(2\right)}\left(0\right)=0.21\pm 0.06$ [18]. Beyond ${\text{WSe}}_{2}$, other monolayer TMDCs such as ${\text{MoSe}}_{2}$ and ${\text{MoS}}_{2}$ have also emerged as promising quantum emitters, exhibiting localized SPE from strain‐ or defect‐induced potential wells. Site‐controlled quantum emitters in monolayer ${\text{MoSe}}_{2}$ have been realized through nanoscale strain confinement [19], whereas defect‐engineered ${\text{MoS}}_{2}$ emitters created through focused helium‐ion irradiation and encapsulated in hBN have shown stable photon antibunching [20]. However, challenges in bare monolayer TMDCs at room temperature include strong exciton–phonon interactions and inefficient light extraction. At 300 K, the exciton linewidth in TMDC monolayers broadens to tens of meV due to phonon‐induced dephasing and scattering, washing out the zero‐phonon line and severely limiting the coherence time of the exciton transition [21]. In a bare monolayer, spontaneous emission is also not directed into a specific optical mode, so most photons radiate into a wide solid angle, resulting in low collection efficiency. To improve this, it has been demonstrated that by deterministically coupling ${\text{WSe}}_{2}$ emitters to gold gap‐plasmon nanocavities, the measured cavity‐enhanced quantum yield jumps from ∼1% to as high as 65% (average 44%) [22]. Even with cavity‐enhanced quantum yields up to 65%, the emission remains limited to cryogenic temperatures, as nonradiative channels and spectral diffusion dominate at higher temperatures. Moreover, it should be noted that despite the enhancement in spontaneous emission rates facilitated by QE–cavity systems, a significant challenge arises from photon bunching. This phenomenon can occur in the absence of strong photon blockade, when the cavity mode allows multiphoton occupancy, thereby increasing the probability of multiphoton emission [8]. To mitigate photon bunching and promote SPE with high purity, spatially aligning quantum emitters, such as TMDC monolayers, with photonic/plasmonic cavities can enable strong light–matter coupling, leading to photon blockade [23].

Despite advances, realizing room‐temperature SPSs with high purity remains an outstanding challenge, motivating novel approaches in materials and device design. More recently, to address the operating temperature issue, Do et al. demonstrated room‐temperature strong coupling between carbon‐based SPEs in a few‐layer hBN film and a high‐Q bound‐state‐in‐the‐continuum (BIC) cavity. This platform achieved a Rabi splitting of ∼4meV with $g^{\left(2\right)}\left(0\right)=0.28$, resulting in an emission purity of $1-g^{\left(2\right)}\left(0\right)\;\approx \;72\%$ [10]. Although the photon antibunching was confirmed in the strong coupling regime at room temperature, the oscillator strength of hBN resulted in a much lower Rabi splitting. This, in turn, led to reduced anharmonicity and decreased photon purity. Additionally, another research group has sought to demonstrate antibunching in photons emitted from a strongly coupled single‐QD‐pillar microcavity system at low temperature [24]. However, due to the relatively small Purcell factor of 61±7, the measured $g^{\left(2\right)}\left(0\right)$ was =0.18. Therefore, it is evident that the challenges in developing a compact and “ideal” SPE are mainly due to the lack of effective coupling between the emitter and the cavity to enhance anharmonicity and emit photons on demand. In parallel, dielectric metasurfaces and photonic crystals have emerged as a complementary route to SPE because they can support low‐loss and higher Q‐factor than plasmonic nanostructures. These features can reduce dissipative broadening and help preserve optical coherence when coupled with emitters [16, 25, 26]. However, in many dielectric resonators, the optical field is concentrated within the high‐index resonator rather than in air, where the TMDC monolayers can be placed. This can reduce exciton–photon overlap, leading to reduced light–matter interaction in the hybrid structures. In this regard, our previous work demonstrated a lithium niobate metasurface with guided‐mode‐resonance platforms coupled to TMDC monolayers, where the Rabi splitting was lower than that achieved with plasmonic metasurface‐TMDC platforms [27, 28].

To address this question, we designed and theoretically investigated a miniaturized SPS by coupling TMDC monolayer emitters to a subwavelength plasmonic Au metasurface. Additionally, the resonant Au metasurface exhibits a tunable Fano‐shaped resonance that can be adjusted by varying its design parameters, which is crucial for aligning the plasmonic mode with the exciton mode of ${\text{WSe}}_{2}$ and other TMDC monolayers [28]. The designed Au metasurface, hereafter referred to as the Au cavity, supports an electric quadrupole (EQ) mode with a higher Q‐factor, facilitating strong coupling with ${\text{MoS}}_{2}$, ${\text{WSe}}_{2}$, and ${\text{MoSe}}_{2}$ monolayers at room temperature. These components are essential for realizing ultra‐compact SPS devices operating in the strong coupling regime, as they enhance the system's anharmonicity. This anharmonicity can be exploited to achieve the photon‐blockade regime, thereby enabling precise on‐demand control over the number of photons transmitted under resonant excitation. As a result, we initially examined the optical properties of the Au metasurface using multipole analysis and discussed its applications after coupling to ${\text{WSe}}_{2}$, ${\text{MoS}}_{2}$, and ${\text{MoSe}}_{2}$. Strong coupling was systematically investigated by varying metasurface thickness, air‐gap width (gap distance), and periodicity [27, 29].

The results indicate that significant Rabi splitting, exceeding 110 meV, can be achieved in Au‐TMDC hybrid nanostructures by optimizing the Au nanostructure parameters. This substantial Rabi splitting is attributed to the higher Q‐factor of the EQ mode induced by the Au cavity. This strong coupling regime at room temperature induces pronounced anharmonicity, resulting in high‐purity SPE from the Au–${\text{WSe}}_{2}$ system with $g^{\left(2\right)}\left(0\right)=4.0\times 10^{-4}$ through CPB. Furthermore, applying the UnPB to the strongly coupled Au–TMDC systems at a drive amplitude of $\Omega_{p}=0.5\;meV$ yields even higher photon purity, with $g^{\left(2\right)}\left(0\right)=1.64\times 10^{-4}$ for Au–${\text{WSe}}_{2}$, $2.30\times 10^{-4}$ for Au–${\text{MoS}}_{2}$, and $1.59\times 10^{-4}$ for Au–${\text{MoSe}}_{2})$. The purity of the emitted photons in these hybrid systems increases (i.e., the $g^{\left(2\right)}\left(0\right)$ value decreases) as the drive strength is reduced. Comparing our results with bare TMDCs emitters, TMDC‐coupled to dielectric microcavities, and plasmonic nanocavity‐${\text{WSe}}_{2}$, our design offers a scalable field enhancement, provides improved TMDC emitter‐cavity overlap, and enables efficient photon extraction [22]. Our results highlight the potential of Au cavity‐TMDCs to realize scalable room‐temperature SPSs with near‐unity purity, thereby opening avenues for integrated quantum photonic devices, including on‐chip quantum networks and secure quantum communications.

## 2 Numerical Simulation and Modeling

### 2.1 ANSYS Optica Results

To investigate SPE in the strong coupling regime, we first need to calculate the optical properties of the uncoupled Au metasurface and TMDC monolayers using finite‐difference time‐domain (FDTD) software (ANSYS Optical Inc.). As a result, a plane wave source with normal incidence along the *z*‐direction, with polarization aligned along the *y*‐direction (90°) was applied. To model an infinite periodic nanostructure, periodic boundary conditions were imposed along the *x*‐ and *y*‐directions. A perfectly matched layer (PML) was applied along the *z*‐direction, with optimized absorption parameters to suppress outgoing wave reflections effectively [28, 29]. As a result, the permittivity value for gold was obtained from data published by Johnson and Christy [30]. Additionally, the exciton resonances were modeled with a thickness of 0.7 nm, and their in‐plane permittivity was described using the Lorentz oscillator [31].

(1) $$\varepsilon \left(E\right)=\varepsilon_{B}+\frac{f}{E_{\text{exciton}}^{2}-E^{2}-i\Gamma E}$$

where $\varepsilon_{B}$ is the background dielectric constant, f is the oscillator strength, $E_{\text{exciton}}$ is the exciton energy, and Γ is the width of the exciton resonances. The modeling parameters for the given TMDC monolayers were set as follows: ${MoS}_{2}$: $\varepsilon_{B}=18$, $f=1.7\;{\text{eV}}^{2}$, $E_{\text{exciton}}=1.86$ eV, and Γ=40 meV, ${WSe}_{2}$: $\varepsilon_{B}=25$, $f=0.7\;{\text{eV}}^{2}$, $E_{\text{exciton}}=1.73$ eV, and Γ=11.4 meV, and ${MoSe}_{2}$: $\varepsilon_{B}=26$, $f=0.98\;{\text{eV}}^{2}$, $E_{\text{exciton}}=1.65$ eV, and Γ=18 meV [27, 32].

### 2.2 Modeling Single Photon Purity

After numerically calculating the Au cavity decay rate $\Gamma_{P}$, emitter decay rate $\Gamma_{X}$, cavity frequency $\omega_{c}$, exciton frequency $\omega_{x}$, and extracting coupling strength (g), the SPS purity was modeled using the quantum toolbox in Python (QuTiP) [33]. Thus, the interaction in coupled Au–TMDCs can be described using the Jaynes–Cummings (JCs) Hamiltonian [34].

(2) $$H_{\text{JC}}=\omega_{c}\;a^{†}a+\omega_{x}\;\sigma^{+}\sigma^{-}+g\left(a\;\sigma^{+}+a^{†}\sigma^{-}\right),$$

where ℏ=1; $a\;\left(a^{†}\right)$ are the bosonic annihilation (creation) operators of the cavity photon mode, and $\sigma^{-}=|g\rangle \;\langle e|$, $\sigma^{+}=|e\rangle \;\langle g|$ are the TMDC emitters lowering and raising operators. Here, we consider a weak drive on the cavity with amplitude $\Omega_{p}$ at frequency $\omega_{p}$, and a weak drive on the emitter (dipole) with amplitude $\Omega_{d}$ at frequency $\omega_{d}$. The lab‐frame Hamiltonian is then expressed as follows:

(3) $$H\left(t\right)=H_{\text{JC}}+\Omega_{p}\;e^{-i\omega_{p}t}\;a^{†}+\Omega_{p}^{*}\;e^{+i\omega_{p}t}\;a+\Omega_{d}\;e^{-i\omega_{d}t}\;\sigma^{+}+\Omega_{d}^{*}\;e^{+i\omega_{d}t}\;\sigma^{-},$$

where $H_{\text{JC}}$ is the JC Hamiltonian. Here, to remove the fast optical oscillations, we move to a rotating frame defined by the unitary

$$U\left(t\right)=\exp\left(i\omega_{p}t\;N\right),\;\text{with}\;N=a^{†}a+\sigma^{+}\sigma^{-}.$$

In this frame, the transformed Hamiltonian is given by the following equation:

$$H^{\prime }\left(t\right)=UHU^{†}-i\;U\frac{\partial U^{†}}{\partial t}.$$

Under this transformation, the operators acquire phase factors, $a\to a\;e^{-i\omega_{p}t}$ and $\sigma^{-}\to \sigma^{-}\;e^{-i\omega_{p}t}$. The resulting Hamiltonian in the rotating frame can be expressed as follows:

(4) $$\begin{array}{ll} H^{\prime }\left(t\right) & =\Delta_{c}\;a^{†}a+\Delta_{x}\;\sigma^{+}\sigma^{-}+g\left(a\sigma^{+}+a^{†}\sigma^{-}\right)+\Omega_{p}a^{†}+\Omega_{p}^{*}a\\ & \;+\Omega_{d}\;e^{-i\Delta_{d}t}\;\sigma^{+}+\Omega_{d}^{*}\;e^{+i\Delta_{d}t}\;\sigma^{-}, \end{array}$$

where the detunings are

$$\Delta_{c}=\omega_{c}-\omega_{p},\;\Delta_{x}=\omega_{x}-\omega_{p},\;\Delta_{d}=\omega_{d}-\omega_{p}.$$

Combining all transformed terms, the rotating‐frame Hamiltonian can be written as follows [35]:

(5) $$\begin{array}{ll} H_{\text{rot}}\left(t\right) & =\Delta_{c}\;a^{†}a+\Delta_{x}\;\sigma^{+}\sigma^{-}+g\left(a\;\sigma^{+}+a^{†}\sigma^{-}\right)\\ & \;+\Omega_{c}\;a^{†}+\Omega_{c}^{*}\;a+\Omega_{x}\;e^{-i\Delta_{d}t}\;\sigma^{+}+\Omega_{x}^{*}\;e^{+i\Delta_{d}t}\;\sigma^{-}, \end{array}$$

with $\Omega_{c}\;\equiv \;\Omega_{p}$ and $\Omega_{x}\;\equiv \;\Omega_{d}$. In this frame, the operators transform as $UaU^{†}=e^{-i\omega_{p}t}a$ and $U\sigma^{\pm }U^{†}=e^{\pm i\omega_{p}t}\sigma^{\pm }$, ensuring that the drive terms become slowly varying (or static under resonant driving).

In the single‐frequency case, where both the cavity and emitter are driven by the same laser $\left(\omega_{d}=\omega_{p}\right)$, the atomic‐drive detuning vanishes $\left(\Delta_{d}=0\right)$ and the Hamiltonian becomes time independent.

(6) $$H=\Delta_{c}\;a^{†}a+\Delta_{x}\;\sigma^{+}\sigma^{-}+g\left(a\;\sigma^{+}+a^{†}\sigma^{-}\right)+\left(\Omega_{c}\;a^{†}+\Omega_{c}^{*}\;a\right)+\left(\Omega_{x}\;\sigma^{+}+\Omega_{x}^{*}\;\sigma^{-}\right).$$

The cavity–emitter system is then described by a density matrix ρ(t) whose evolution is governed by the quantum master equation.

(7) $$\dot{\rho }=-i\;\left[H,\rho \right]+D\left[\sqrt{\Gamma_{P}}\;a\right]\rho +D\left[\sqrt{\Gamma_{X}}\;\sigma^{-}\right]\rho ,$$

with Lindblad dissipator

(8) $$D\left[o\right]\rho =o\rho \;o^{†}-\frac{1}{2}\;o^{†}o\;\rho -\frac{1}{2}\;\rho \;o^{†}o.$$

We focus on the steady‐state solution, ∂ρ/∂t=0, and obtain numerical results by solving Equations (7) and (8) in QuTiP [33], truncating the cavity Hilbert space to dimension six. The single‐photon purity is then verified using the steady‐state zero‐delay second‐order correlation.

(9) $$g^{\left(2\right)}\left(0\right)=\frac{\langle {\hat{a}}^{†}{\hat{a}}^{†}\hat{a}\;{\hat{a}\rangle }_{\text{ss}}}{{\left(\langle {\hat{a}}^{†}{\hat{a}\rangle }_{\text{ss}}\right)}^{2}}\;.$$

## 3 Result and Discussion

### 3.1 Multipole Analysis of Au Metasurface

To reveal the physical nature of the designed Au metasurface (cavity), multipole decompositions were performed across a range of design parameters. Subsequently, to analyze the light scattering from the metasurface illustrated in Figure 1a, a Cartesian multipole decomposition was applied. The scattered power was obtained from the Cartesian multipole expansion by integrating the current–density distribution J within the metasurface unit cell (see Figure 1b and Supporting Information S1: Section S1) [36, 37]. As can be seen from Figure 1d–f, the incident electric field excites an electric–dipole (ED) mode, producing a broadband scattering spectrum centered near 500nm. Because ED modes couple efficiently to the far field, they exhibit intrinsically low quality (Q) factors and therefore broad absorption bands. Additionally, this ED response diminishes, and a dominant electric–quadrupole (EQ) resonance emerges at longer wavelengths (660–730nm). Regardless of thickness and/or slit gap, the EQ mode becomes predominant, whereas the contributions from magnetic dipole (MD), magnetic quadrupole (MQ), and toroidal dipole (TD) remain minimal. Near–field intensity maps (Figure 1c) verify that EQ‐dominant regimes confine energy within the meta–atom, suppressing radiative leakage and leading to narrow high‐Q absorption features. Because quadrupolar radiation is two orders higher in the multipole hierarchy, it couples only weakly to the far field, further reducing radiation losses. This suppression enhances the Q‐factor, which could compensate for Ohmic losses in gold by prolonging the dwell time of energy in near‐field hotspots. As shown in Figure 1g–i, the resulting EQ‐dominated absorption spectra provide clear advantages for light–matter interaction.

**FIGURE 1:**
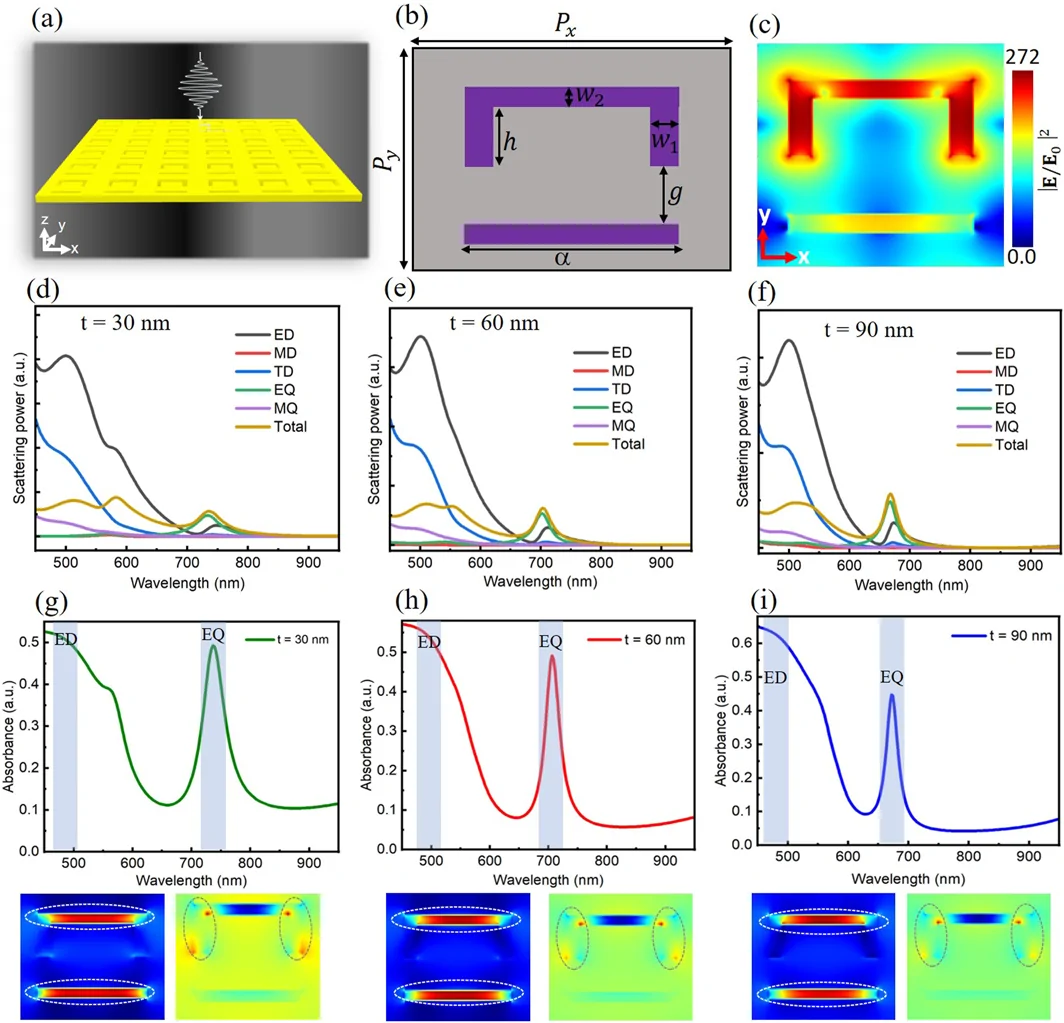
Multipole analysis and absorption spectra of Au metasurface. (a) Schematic drawing of the metasurface. (b) Top views of a unit cell of Au metasurface with design parameters of $W_{1}=39$ nm, $W_{2}=33$ nm, h=106 nm, g=106 nm, α=307 nm, and $P_{x}=P_{y}$ = 420 nm. (c) Near‐field enhancement of the unit cell. (d–f) Multipole analysis of Au metasurface with (d) t=30 nm, (e) t=60 nm, and (f) t=90 nm. The corresponding multipole expansion equations are found in Supporting Information S1: section S1. (g–i) The corresponding absorption spectra of the Au metasurface of the multipole decomposition in (d–f). The ED excited by incident light produced a broad spectrum around 500 nm, whereas the higher‐EQ mode induced narrow spectra (see their corresponding field profiles (g–i)).

The metasurface optical properties, such as transmittance, reflectance, and absorbance, dominated by the EQ, are tunable by varying the thickness and slit widths of the structure (see Figure 2a–c). Additionally, electric field confinement by the metasurface is strongly dependent on its design parameters. For instance, as shown in Figure 1c, the near‐field enhancement $\left({\left|E/E_{0}\right|}^{2}\right)$ of the uncoupled Au cavity, which reaches as high as 272 at a thickness of 42.3 nm, decreases with increasing thickness and slit width [28]. This is because a smaller metasurface thickness results in a reduced skin depth, meaning that the electromagnetic fields are more tightly confined near its surface, thereby enhancing the interaction with TMDC emitters. The plasmon resonance of the Au cavity, shown in Figure 2b, reveals a redshift when decreasing its thickness, indicating the presence of strong plasmon coupling and higher field confinement [38]. This tunability is essential for aligning the plasmon resonance with the exciton transitions in TMDC monolayers. As a result, varying the design parameters enables investigation of plasmon–exciton coupling in Au–${\text{WSe}}_{2}$ heterostructures. For instance, as shown in Figure 2d,f, the plasmon resonance aligns with the exciton resonance of the ${\text{WSe}}_{2}$ monolayer. Matching these resonances is crucial for achieving strong plasmon‐exciton coupling [28].

**FIGURE 2:**
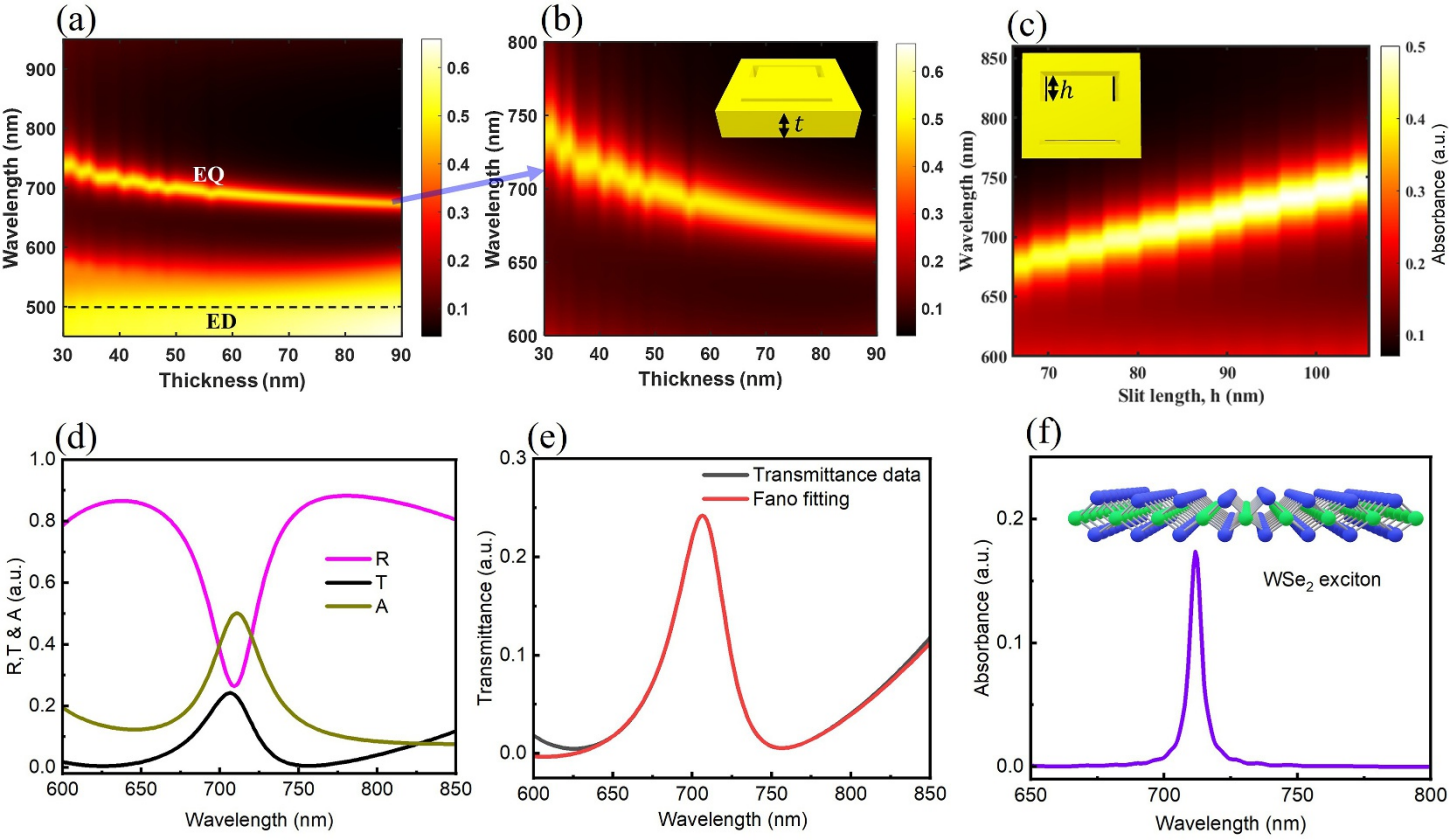
Tuning plasmon resonances dominated by EQ. (a–b) Absorbance mapping of Au metasurface with varying thickness, keeping other parameters as in Figure 1b. (c) Absorbance mapping of Au metasurface with varying gap length (h). (d) Reflectance (*R*), transmittance (*T*), and absorbance (*A*) of the uncoupled Au cavity matched with the ${\text{WSe}}_{2}$ monolayer. (e) Induced Fano‐shaped transmission spectra fitted using Equation (11). (f) Absorbance spectrum of ${\text{WSe}}_{2}$ monolayer modeled using Equation (1).

Furthermore, it is worth mentioning that, as can be seen from Figure 2e, the transmission spectrum exhibits Fano‐shaped resonances [39], which are sensitive, tunable, and applicable when coupled with TMDC emitters. This Fano resonance could arise from a broad (bright) mode of the ED and a narrow (dark) mode of the EQ in the Au metasurface (see Figure 1d–e). To confirm this, we fitted the numerical data of the transmittance using the Fano formula for metasurface [27, 40].

(10) $$T\left(\omega \right)=T_{bg}+T_{0}\left(\frac{{\left[q+\frac{2\left(\omega -\omega_{0}\right)}{\Gamma_{p}}\right]}^{2}}{1+{\left[\frac{2\left(\omega -\omega_{0}\right)}{\Gamma_{p}}\right]}^{2}}\right)$$

where $T_{bg}$ and $T_{0}$ denote the constant background and the resonance amplitude, respectively; $\Gamma_{p}$ is the linewidth of the Au metasurface; and q is the Fano asymmetry parameter quantifying the interference between bright (continuum) and dark (discrete) channels. Equation (10) is appropriate when the background is flat. For the present plasmonic Au cavity, dispersive loss channels produce a slowly varying baseline, so we extend the $T_{bg}$ to capture the full response.

(11) $$T\left(\omega \right)=\underset{\text{slow}\;\text{background}}{\underbrace{C_{0}+C_{1}\left(\omega -\omega_{0}\right)+C_{2}{\left(\omega -\omega_{0}\right)}^{2}}}+\;T_{0}\left(\frac{{\left(q+\frac{2\left(\omega -\omega_{0}\right)}{\Gamma_{p}}\right)}^{2}}{1+{\left(\frac{2\left(\omega -\omega_{0}\right)}{\Gamma_{p}}\right)}^{2}}\right)$$

where, $C_{0}$, $C_{1}$, and $C_{2}$ are fitting parameters. As a result, the transmission spectra shown in Figure 1d–f confirm that the quadrupole mode constitutes the resonant state, whereas the dipole mode provides the nonresonant background, forming a Fano‐shaped resonance, and it's consistent across different Au thicknesses (see Figure 1d–f).

### 3.2 Strong Coupling in Au‐${\text{WSe}}_{2}$

To investigate strong coupling in Au‐TMDCs and apply it to SPE applications, we began by combining the high quality factor and low volume of Au cavities with a ${\text{WSe}}_{2}$ monolayer. This regime fosters the formation of hybrid states that are part light and part matter, have a small effective mass, and are faster. Therefore, to investigate their coupling strength, a hybrid structure comprising a plasmonic Au cavity and an excitonic ${\text{WSe}}_{2}$ monolayer was designed as shown in Figure 3a. The corresponding energy resonances are observed in Figure 3c, demonstrating that the coupling strength can be effectively modulated by varying the thickness of the Au cavity. A thinner thickness, corresponding to a smaller skin depth and higher field confinement, leads to stronger interactions [29]. The near‐field enhancement in Au‐${\text{WSe}}_{2}$ structures shown in Figure 3b with ${\left|E/E_{0}\right|}^{2}=2040$, is higher than of uncoupled Au cavity, which exhibits ${\left|E/E_{0}\right|}^{2}=272$ in Figure 1c. This indicates enhanced light–matter interaction in the hybrid system. To quantify the plasmon‐exciton interaction strength, we calculated the coupling strength by fitting the resonance energy of the upper polariton (UP) and lower polariton (LP) based on the coupled oscillator model shown in Figure 3d [28, 41].

(12) $$\left(\begin{array}{cc} E_{P}+i\hbar \Gamma_{P} & g\\ g & E_{X}+i\hbar \Gamma_{X} \end{array}\right)\left(\begin{array}{c} \alpha \\ \beta \end{array}\right)=E_{\pm }\left(\begin{array}{c} \alpha \\ \beta \end{array}\right)$$

where $E_{P}\;\left(\omega_{c}\right)$ and $E_{P}\;\left(\omega_{x}\right)$ are the resonance energies of the plasmon mode of Au cavity and the exciton mode of ${WSe}_{2}$, respectively; g is the coupling strength between photons and excitons; and $\hbar \Gamma_{P}$ and $\hbar \Gamma_{X}$ are the half width at half maximum (HWHM) of the plasmonic mode and exciton modes. α and β are Hopfield coefficients, which are used to describe the weight of the photonic and exciton modes, satisfying ${\left|\alpha \right|}^{2}+{\left|\beta \right|}^{2}=1$ (see Figure 3d). As a result, by solving Equation (12), the eigenvalues $E_{\pm }$, which correspond to the energies of the new quasiparticles, can be calculated as follows:

(13) $$E_{\pm }=\frac{1}{2}\left[\left(E_{X}+E_{P}\right)+i\left(\hbar \Gamma_{X}+\hbar \Gamma_{P}\right)\right]\pm \sqrt{g^{2}-\frac{1}{4}{\left[\left(E_{X}-E_{P}\right)+i\left(\hbar \Gamma_{X}-\hbar \Gamma_{P}\right)\right]}^{2}}.$$

The Rabi splitting (ℏΩ) of the exciton‐plasmon is then extracted from the absorbance mapping data of Figure 3c at the detuning $\left(E_{P}-E_{X}=0\right)$, and Equation (13) can be expressed as follows:

(14) $$\hbar \Omega =E_{+}-E_{-}=2\sqrt{g^{2}-\frac{1}{4}{\left(\hbar \Gamma_{X}-\hbar \Gamma_{P}\right)}^{2}}.$$

After extracting ℏΩ from 3(c) and substituting values of $\hbar \Gamma_{P}$ and $\hbar \Gamma_{X}$ into Equation (14), the coupling strength (g) is subsequently determined to be 44.34  meV. As shown in Figure 3d, the dispersion relation is then fitted as a function of Au thickness. This confirms that the values ℏΩ extracted from 3c at zero detuning is consistent with 2g obtained from the fitted disperssion in Figure 3d. The Rabi splitting of the system can exceed 100 meV by decreasing the Au thickness to 50 nm (see Figure 3f and Supporting Information S1: Figure S2 in the supplementary information). According to Equation (14), the calculated Rabi splitting must satisfy two conditions to ensure the system exhibits strong coupling [41].

(15) $$g>\frac{\left|\hbar \Gamma_{P}-\hbar \Gamma_{X}\right|}{2}\;\text{and}\;g>\sqrt{\frac{1}{2}{\left(\hbar \Gamma_{P}\right)}^{2}+\frac{1}{2}{\left(\hbar \Gamma_{X}\right)}^{2}}.$$

**FIGURE 3:**
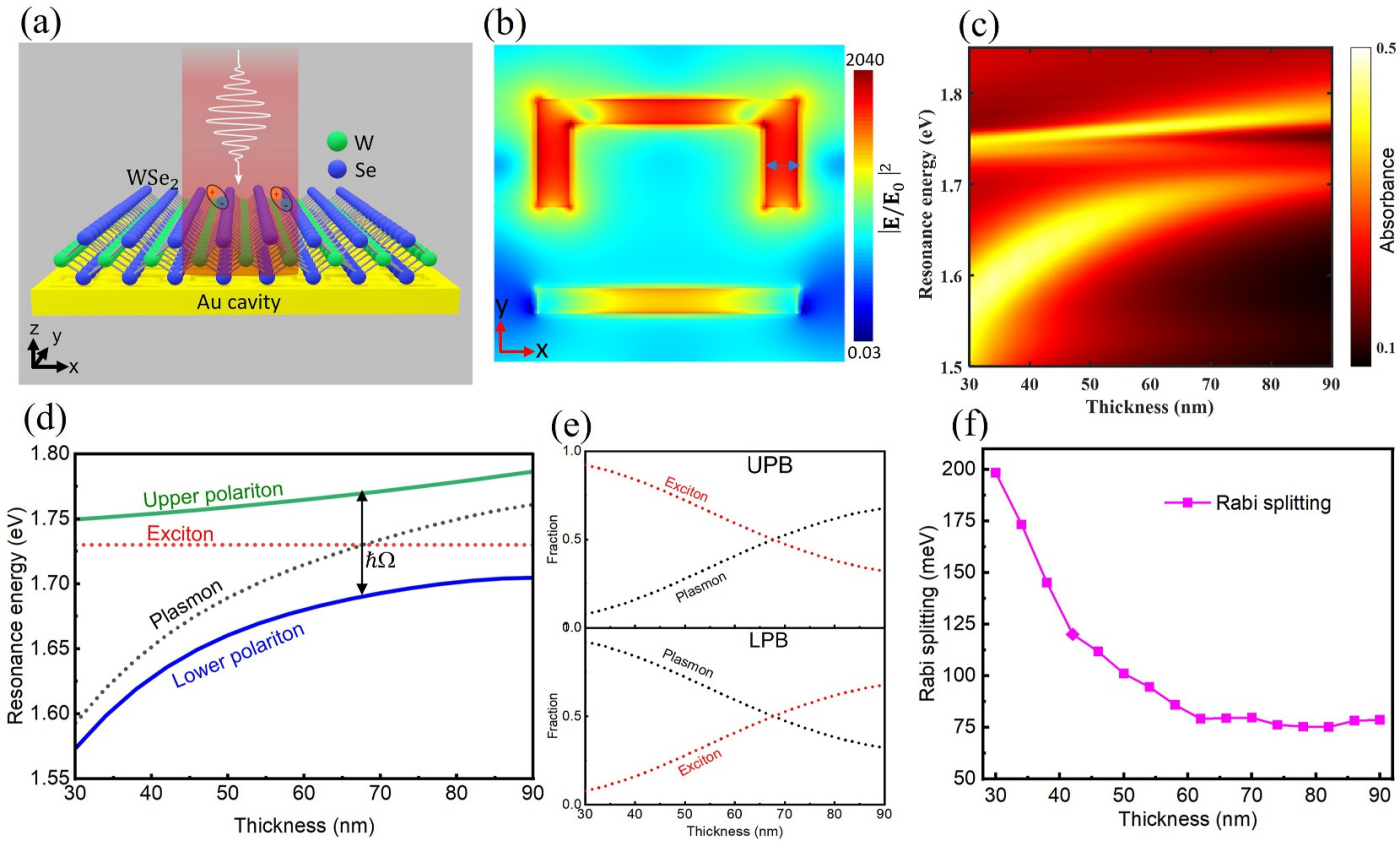
Strong plasmon–exciton coupling in Au–${\text{WSe}}_{2}$. (a) Schematic showing ${\text{WSe}}_{2}$ atop an Au cavity at normal incidence. (b, c) Absorption spectrum mapping of Au‐${\text{WSe}}_{2}$ with varying Au thickness from 30 to 90 nm while keeping other parameters constant ($W_{1}=39$ nm, $W_{2}=33$ nm, h=106 nm, g=106 nm, α=307 nm, and p=420 nm. (d) Dispersion curves of Au‐${\text{WSe}}_{2}$ as a function of thickness, extracted from the absorption mapping in panel (b). Here, the polariton branches at which the uncoupled Au metasurface and ${\text{WSe}}_{2}$ exciton induced a larger Rabi splitting of greater than 100 meV (see Supporting Information S1: Figure S2). (e) Hopfield coefficients showing exciton and plasmon contributions to UP and LP as a function of Au thickness. (f) Thickness‐dependent Rabi splitting of Au–${\text{WSe}}_{2}$, with other parameters identical to panel (a). Thinner Au metasurfaces lead to stronger field confinement and greater Rabi splitting due to a smaller skin depth.

As a result, by substituting the values of $\hbar \Gamma_{P}$ with 43.6 meV and $\hbar \Gamma_{X}$ with 5.7 meV into Equation (15), the system satisfied both conditions simultaneously. Furthermore, as shown in Figure 3e, the coupling strength is larger for a thinner Au nanostructure due to stronger field confinement. The results confirm that the coupling between the Au cavity and ${\text{WSe}}_{2}$ excitons belongs to the strong coupling regime. This strong coupling regime with pure detuning is crucial for achieving antibunching through photon blockade.

### 3.3 Photon‐Blockade in Au‐${\text{WSe}}_{2}$

The demonstrated strong coupling in the Au cavity–${WSe}_{2}$ monolayer, with large Rabi splitting shown in Figure 3, reveals that the system is highly anharmonic [42]. Hence, the strongly coupled Au cavity interacting with a monolayer ${\text{WSe}}_{2}$ exciton can give rise to pronounced quantum nonlinearities, enabling conventional photon blockade (CPB). To implement and dynamically manipulate PB in this strongly coupled system, we apply weak driving fields to the cavity in Equation (6).

(16) $$H=\Delta_{c}\;a^{†}a+\Delta_{x}\;\sigma^{+}\sigma^{-}+g\left(a\;\sigma^{+}+a^{†}\sigma^{-}\right)+\left(\Omega_{c}\;a^{†}+\Omega_{c}^{*}\;a\right).$$

CPB is a quantum nonlinear effect in which the absorption of a single photon by an Au cavity prevents the entry of a second. This phenomenon originates from the anharmonicity of the JCs energy ladder in a strongly coupled Au–${\text{WSe}}_{2}$ system. As can be seen from Figure 4a, once the first rung is occupied, the higher rungs are energetically shifted by an amount set by g, preventing resonant re‐excitation and giving rise to CPB. When the first photon is resonantly absorbed, it shifts the effective resonance frequency for the second photon, effectively blocking the cavity and enforcing the emission of photons one by one (see Figure 3a). This is a direct consequence of a two‐level system strongly coupled to a cavity, which gives rise to polaritonic dressed states and the photon blockade effect [43]. This, in turn, results in strongly antibunched emission.

**FIGURE 4:**
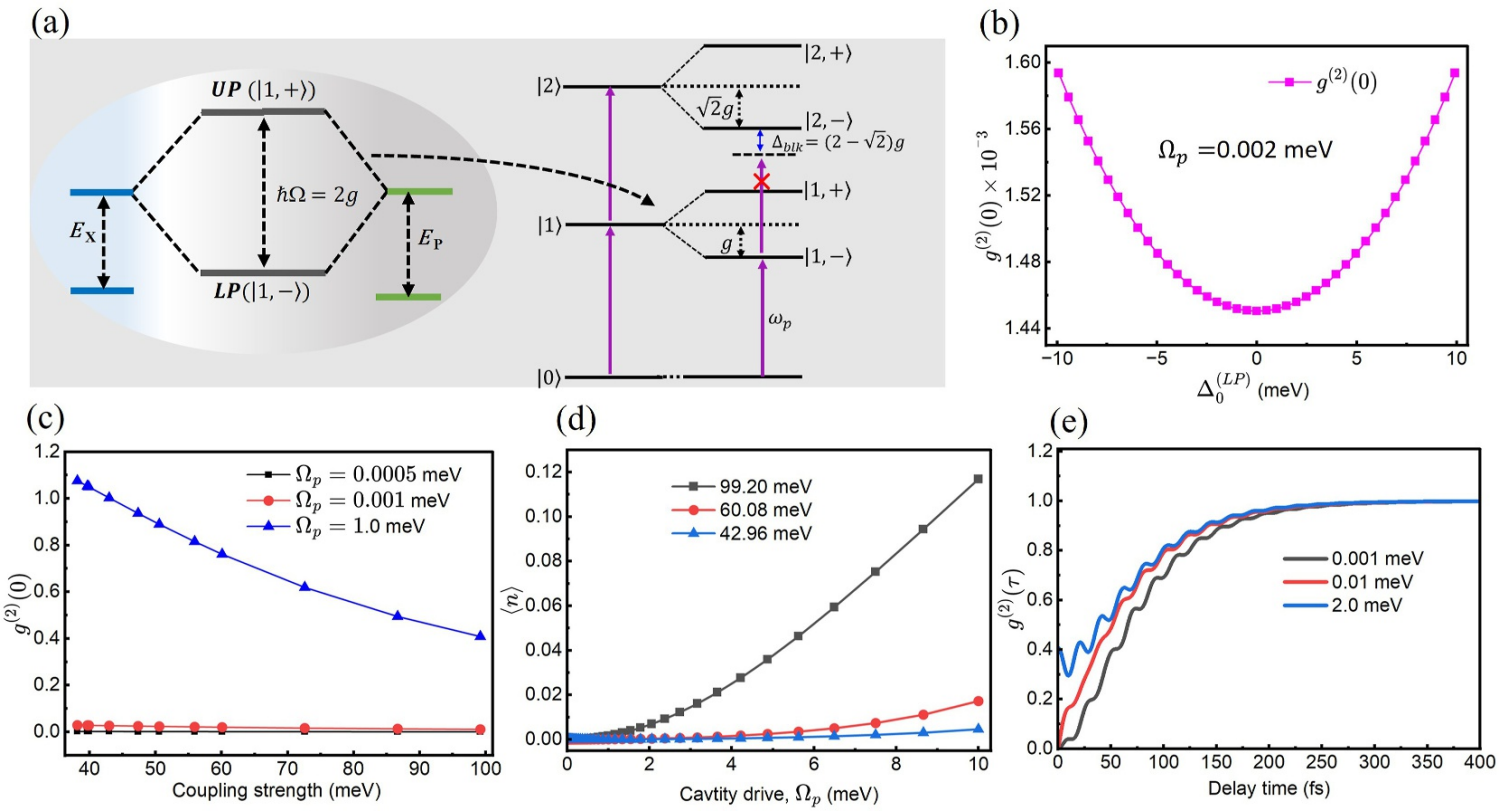
Single‐photon purity in Au‐${\text{WSe}}_{2}$ through CPB (a) energy‐level diagram of a two‐level quantum emitter resonantly coupled to a plasmonic cavity field with coupling strength g. Two‐photon emission is suppressed by the anharmonic JCs ladder, realizing PB. (b) Calculated $g^{\left(2\right)}\left(0\right)$ versus the detuning $\Delta_{0}^{\left(LP\right)}$, where $\Delta_{0}^{\left(LP\right)}\approx -g$ corresponds to the LP resonance. (c) $g^{\left(2\right)}\left(0\right)$ versus coupling strength g for several cavity‐drive amplitudes, $\Omega_{p}=0.001\;meV$, 0.002meV, and 2.0meV. Increasing g enhances the JC anharmonicity $\Delta_{\text{blk}}=\left(2-\sqrt{2}\right)g$, detuning the two‐photon transition and reducing $g^{\left(2\right)}\left(0\right)$. (d) Mean intracavity photon number 〈n〉 versus drive amplitude $\Omega_{p}$ for coupling strengths of g=99.20meV, 60.08meV, and 42.96meV. (e) $g^{\left(2\right)}\left(\tau \right)$ versus delay time τ at fixed coupling strength.

Subsequently, the proposed hybrid system, an Au cavity strongly coupled to a monolayer of ${\text{WSe}}_{2}$, is exceptionally well suited for realizing CPB at room temperature. The key figure of merit here is the light–matter coupling strength g, which in our system reaches tens of meV. This leads to a high cooperativity $C=4g^{2}/\left(\Gamma_{P}\Gamma_{X}\right)>11$, placing the system firmly in the strong coupling regime required for CPB. Numerical demonstrations of Rabi splittings exceeding 100 meV in similar plasmon–TMDC systems [28] confirm that robust room‐temperature operation is feasible. Moreover, the giant optical nonlinearities in ${\text{WSe}}_{2}$ directly strengthen the intrinsic nonlinearity that drives the blockade.

To quantitatively describe CPB, we numerically solve the dissipative JCs Hamiltonian [Equation 16]. When the cavity is resonantly driven at the lower polariton frequency $\left(\omega_{p}\;\approx \;\omega_{c}-g\right)$, the system exhibits a pronounced photon‐blockade signature. This effect arises from the anharmonicity of the JCs ladder, $\Delta_{\text{blk}}=\left(2-\sqrt{2}\right)g$ (see Supporting Information S1: Section S2 for detailed procedures) [44]. In this regime, the single‐photon transition |g,0〉→|1,−〉 is resonant, whereas the two‐photon transition |1,−〉→|2,−〉 is detuned by $\Delta_{\text{blk}}$, thereby suppressing double excitation and yielding high‐purity antibunched emission (see Figure 4a). As shown in Figure 4b, the anharmonic energy spacing forcefully suppresses the two‐photon probability, producing a deep minimum of $g^{\left(2\right)}\left(0\right)=1.4\times 10^{-3}$ at a drive amplitude of $\Omega_{p}=0.002\;meV$. This pronounced dip near the lower polariton detuning marks the regime of highest single‐photon purity, arising from photon blockade in the strong coupling regime. Here, the anharmonic energy ladder of the hybrid exciton–photon system prevents simultaneous occupation of multiple cavity photons, yielding distinctly sub‐Poissonian statistics. At resonance with the lower polariton, the exciton–photon coupling is maximized, enabling efficient SPE.

As demonstrated in Figure 3e, increasing the Au cavity thickness causes a decrease in the Rabi splitting [28]. This, in turn, reduces the anharmonicity of the hybrid system, resulting in larger $g^{\left(2\right)}\left(0\right)$ and lower purity (see Figure 4c). Figure 4c,d further illustrate the dependence of CPB on coupling strength and drive amplitude. In Figure 4c, $g^{\left(2\right)}\left(0\right)$ decreases systematically with increasing g, because the larger anharmonicity $\Delta_{\text{blk}}$ increasingly detunes the two‐photon transition and strengthens the blockade. At a coupling strength of g=99.2meV and under weak driving, where $\Omega_{p}$ is much smaller than both $\Delta_{\text{blk}}$ and the dissipation rates, the system exhibits nearly ideal antibunching with $g^{\left(2\right)}\left(0\right)\;\approx \;10^{-4}$, corresponding to near‐unity single‐photon purity [45]. As the drive amplitude increases (red and blue curves), power broadening partially lifts the blockade, enabling excitation of higher JCs rungs and raising $g^{\left(2\right)}\left(0\right)$ toward the classical limit. The complementary behavior is seen in Figure 4d, where the mean intracavity photon number 〈n〉 is plotted as a function of $\Omega_{p}$ for various g. Stronger coupling enhances both the light–matter hybridization and the nonlinear energy shift, resulting in a higher 〈n〉 and improved antibunching at the same drive strength. The demonstrated $g^{\left(2\right)}\left(0\right)$ in strongly coupled Au–${\text{WSe}}_{2}$ is significantly lower than in previously reported weakly coupled systems of gold nanocubes on a sapphire substrate supporting monolayer ${\text{WSe}}_{2}$ (${\text{WSe}}_{2}$/Au/Sapp on an Au mirror), where $g^{\left(2\right)}\left(0\right)=0.15\pm 0.03$ was obtained at low temperature [22].

We now investigate the dynamics of the time‐delayed second‐order correlation function $g^{\left(2\right)}\left(\tau \right)$ (with the equal‐time value given by τ=0). As shown in Figure 4e, the correlation starts from a strongly antibunched value $g^{\left(2\right)}\left(0\right)\ll 1$, a clear signature of photon blockade, and gradually rises toward unity as τ increases. Operationally, this can be calculated through the normalized $g^{\left(2\right)}\left(\tau \right)$ defined as follows:

(17) $$g^{\left(2\right)}\left(\tau \right)=\frac{\langle a^{†}\left(t\right)a^{†}\left(t+\tau \right)a\left(t+\tau \right)a\left(t\right)\rangle }{\langle a^{†}{a\rangle }^{2}},$$

This behavior reflects the transition from quantum correlations at short times to Poissonian statistics at long delays, where subsequent emission events become uncorrelated. The saturation of $g^{\left(2\right)}\left(\tau \right)\to 1$ thus marks the recovery of classical photon statistics, consistent with the finite coherence time of the polariton mode and the dissipative nature of the strongly coupled Au–${\text{WSe}}_{2}$ system.

As a result, the results presented above confirm that the strongly coupled Au–${\text{WSe}}_{2}$ platform exhibits sufficient intrinsic nonlinearity to generate quantum light through CPB at room temperature. Furthermore, the extracted purity, $1-g^{\left(2\right)}\left(0\right)$, is already competitive with leading cryogenic implementations, underscoring the promise of plasmon–exciton hybrid states.

In the current design of a strongly coupled plasmon–exciton platform (Figure 3), CPB provides a quantitative benchmark that establishes the polaritonic ladder and confirms the onset of SPE. Building on this foundation, an analytical expression for the optimal balance between the cavity and emitter drives becomes essential. To this end, we implement a two‐mode UnPB protocol by neglecting the recycling (quantum‐jump) terms $o\;\rho \;o^{†}$ in the weak‐drive/no‐jump approximation. The remaining anticommutator yields an effective non‐Hermitian Hamiltonian $H_{\text{nH}}$ of the following form [35]:

(18) $$H_{\text{nH}}=H-i\frac{\Gamma_{P}}{2}\;a^{†}a-i\frac{\Gamma_{X}}{2}\;\sigma_{+}\sigma_{-},$$

where H denotes the coherent JCs Hamiltonian. Comparing H in Equation (6), defined at the drive frequency with detunings $\Delta_{c}$ (cavity) and $\Delta_{x}$ (emitter), to $H_{\text{nH}}$ in Equation (18) shows that the dissipative contributions appear as diagonal terms in the excitation‐number operators $a^{†}a$ and $\sigma_{+}\sigma_{-}$. Consequently, within the no‐jump picture, one may introduce the complex detunings.

$$\Delta_{c}\;\to \;{\tilde{\Delta }}_{c}\;≔\;\Delta_{c}-i\frac{\Gamma_{P}}{2},\;\Delta_{x}\;\to \;{\tilde{\Delta }}_{x}\;≔\;\Delta_{x}-i\frac{\Gamma_{X}}{2},$$

which yield the effective non‐Hermitian Hamiltonian $H_{\text{nH}}$,

(19) $$H_{\text{nH}}\;\equiv \;H_{\text{eff}}={\tilde{\Delta }}_{c}\;a^{†}a+{\tilde{\Delta }}_{x}\;\sigma_{+}\sigma_{-}+g\;\left(a\;\sigma_{+}+a^{†}\sigma_{-}\right)+H_{\text{drive}}.$$

To analyze the relative phase and amplitude of the cavity‐like and exciton‐like drives, we introduce the basis states |n,α〉, where n denotes the number of excitations in the cavity mode and α∈{g,e} indicates the atomic state (ground or excited). In the weak‐excitation regime, it is sufficient to truncate the Hilbert space to the lowest excitation manifolds (N=2). The system wavefunction can then be expressed as follows:

(20) $$|\psi \left(t\right)\rangle \;\approx \;c_{0g}|0,g\rangle +c_{1g}|1,g\rangle +c_{0e}|0,e\rangle +c_{2g}|2,g\rangle +c_{1e}|1,e\rangle +\text{⋯}\;,$$

where higher‐excitation terms with n≥3 are neglected owing to their negligible population under weak driving. As demonstrated in Supporting Information S1: Section S3, within the truncated excitation basis, the two‐photon amplitude $c_{2g}$ quantifies the probability of occupying the state |2,g〉. Imposing $c_{2g}=0$ yields the following approximate quadratic condition:

(21) $$g^{2}\;\Omega_{d}^{2}\;-\;2g\;{\tilde{\Delta }}_{c}\;\Omega_{p}\Omega_{d}\;+\;\Omega_{p}^{2}\left({\tilde{\Delta }}_{c}{\tilde{\Delta }}_{x}+g^{2}\right)\;≃\;0,$$

where $\tilde{\Delta }\;\equiv \;{\tilde{\Delta }}_{c}+{\tilde{\Delta }}_{x}$. Details of the derivation can be found in Supporting Information S1: Section S3. Solving Equation (21) for the emitter drive yields the following equation:

(22) $$\Omega_{d}^{\left(\pm \right)}=\frac{\Omega_{p}}{g}\;\left(\tilde{\Delta }\pm \sqrt{\tilde{\Delta }\;{\tilde{\Delta }}_{r}-g^{2}}\right),$$

and, fixing the branch of the complex square root consistently with the drive‐phase convention for $\Omega_{p}$,

(23) $$\Omega_{d}^{\left(\pm \right)}=\frac{\Omega_{p}}{g}\;\left(\tilde{\Delta }\pm \text{sgn}\;\left(\Omega_{p}\right)\sqrt{\tilde{\Delta }\;{\tilde{\Delta }}_{r}-g^{2}}\right).$$

This interference‐engineered approach preserves room‐temperature operation and yields deeper antibunching than is accessible with CPB alone. Consequently, in the sweep we vary only the cavity‐drive amplitude $\Omega_{p}$; for each $\Omega_{p}$ we recompute the emitter drive $\Omega_{d}$ from the interference condition derived in the weak‐excitation limit, so that the emitter amplitude and phase co‐tune to maintain cancellation in the two‐photon sector. This procedure yields strong antibunching at low power, with $g^{\left(2\right)}\left(0\right)$ approaching zero (see Figure 5b). Additionally, at a fixed value of $\Omega_{p}=1\;meV$, $g^{\left(2\right)}\left(0\right)$ decreases with increasing coupling strength in Au–${\text{WSe}}_{2}$ (see Figure 5d). As shown in Figure 5b,d, near‐perfect photon blockade is obtained in the weak‐driving regime, as indicated by $g^{\left(2\right)}\left(0\right)\;\ll \;1$. The corresponding mean intracavity photon number $\langle \hat{n}\rangle$, shown in Figure 5c, remains finite but low, reflecting the weak‐driving condition under which the polariton blockade operates. Therefore, rather than assigning a single quantum‐efficiency value from an idealized cavity–emitter expression, we evaluate the device performance using the single‐photon purity, quantified by $g^{\left(2\right)}\left(0\right)$, and the intracavity photon population, quantified by $\langle \hat{n}\rangle$.

**FIGURE 5:**
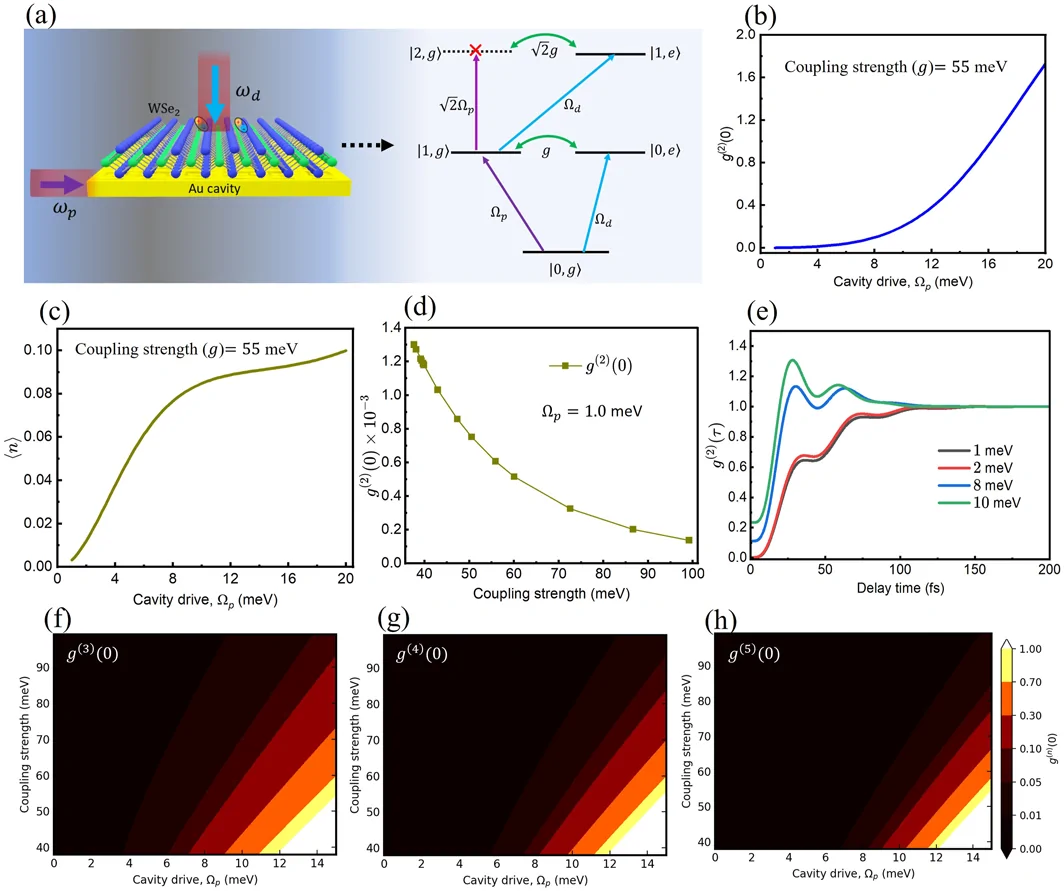
Single‐photon purity in Au–${\text{WSe}}_{2}$ through UnPB. (a) Schematics of the Au–${\text{WSe}}_{2}$ hybrid and energy‐level diagram of Au‐${\text{WSe}}_{2}$, showing UnPB. Imposing $c_{2g}=0$ [Equations (21) and (23)] enforces destructive interference between cavity‐ and emitter‐driven two‐photon pathways, suppressing the two‐photon manifold and yielding strong antibunching (see Supporting Information S1: section S3 and S4 for further explanation). (b, c) $g^{\left(2\right)}\left(0\right)$, and mean intracavity photon number 〈n〉 versus $\Omega_{p}$ at fixed coupling strength of g=55meV. (d) Dependence of $g^{\left(2\right)}\left(0\right)$ on the coupling strength g at a fixed drive $\Omega_{p}=1\;meV$, showing that stronger light–matter coupling enhances the SP purity. (e) $g^{\left(2\right)}\left(\tau \right)$ as a function of τ for $\Omega_{p}=1\;meV$ and g=55meV, exhibiting a pronounced antibunching dip $g^{\left(2\right)}\left(0\right)\;\ll \;1$ and recovery toward unity at large τ. (f–h) Higher‐order equal‐time correlation functions $g^{\left(3\right)}\left(0\right)$, $g^{\left(4\right)}\left(0\right)$, and $g^{\left(5\right)}\left(0\right)$ as functions of g and $\Omega_{p}$ for the two‐drive JC system. Extended dark regions with $g^{\left(n\right)}\left(0\right)\;\ll \;1$ in all three panels demonstrate that, over a broad range of $\left(g,\Omega_{p}\right)$, multiphoton events up to at least the fifth order are strongly suppressed, confirming that the interference‐based two‐drive scheme realizes a near‐ideal SPE.

Furthermore, the time‐resolved correlations $g^{\left(2\right)}\left(\tau \right)$ shown in Figure 5e exhibit a pronounced antibunching dip at τ=0, followed by a gradual recovery to unity, consistent with sequential Single‐photon emission. Together with the steady‐state statistics, these results highlight the advantages of the Au–${\text{WSe}}_{2}$ platform, which combines interference‐based UnPB with a genuine two‐level nonlinearity at room‐temperature‐compatible parameters, thereby simultaneously suppressing two‐photon and higher‐order multiphoton coincidences and providing a practical route toward integrated SPSs.

In addition to investigating the single‐photon purity through $g^{\left(2\right)}\left(0\right)$ in the UnPB regime, we further evaluate higher‐order correlations to assess multiphoton suppression in the two‐drive JCs system. Figure 5f–h show the higher‐order equal‐time correlation functions $g^{\left(3\right)}\left(0\right)$, $g^{\left(4\right)}\left(0\right)$, and $g^{\left(5\right)}\left(0\right)$ as functions of the light–matter coupling strength g and the cavity‐drive amplitude $\Omega_{p}$ for the two‐drive JCs system. These quantities extend the standard equal‐time second‐order correlation $g^{\left(2\right)}\left(0\right)$ to probe multiphoton correlations beyond simple antibunching. In a true SPS, all orders of the correlation functions should satisfy $g^{\left(n\right)}\left(0\right)\ll 1$, indicating a negligible probability of emitting n or more photons at once. The nearly uniform dark regions in Figure 5f–h confirm that, in the two‐drive configuration, multiphoton events up to fifth order are coherently suppressed across a broad parameter range. This behavior originates from destructive quantum interference between the cavity and emitter excitation pathways, thereby eliminating higher‐order photon resonances. As the drive amplitude $\Omega_{p}$ increases, partial breakdown of this interference gradually raises $g^{\left(n\right)}\left(0\right)$, although the suppression remains robust for moderate $\Omega_{p}$ and large g. Evaluating higher‐order correlations up to n=5 thus provides a stringent benchmark for verifying genuine SPE, because a low $g^{\left(2\right)}\left(0\right)$ alone may not rule out residual three‐ or four‐photon bursts. The consistently small values of $g^{\left(3\right)}\left(0\right)$, $g^{\left(4\right)}\left(0\right)$, and $g^{\left(5\right)}\left(0\right)$ shown in Figure 5f–h demonstrates that the two‐drive scheme achieves near‐ideal single‐photon purity, extending photon blockade to higher orders of the correlation hierarchy.

### 3.4 Antibunching in Strongly Coupled Au–${\text{MoS}}_{2}$ and Au‐${\text{MoSe}}_{2}$ System

Building on our Au–${\text{WSe}}_{2}$ baseline for room‐temperature SPE in the strong coupling regime shown in Figures 4, 5, we now examine Au–${\text{MoS}}_{2}$ and Au–${\text{MoSe}}_{2}$, whose tightly bound A excitons at visible energies can be spectrally matched to tunable Au plasmonic modes to form a plasmon–exciton hybrid state satisfying $g\;≳\;\left(\Gamma_{P}+\Gamma_{X}\right)/4$. To enhance the coupling strength and nonlinearity, the plasmon resonance was tuned by varying the design parameters shown in Figure 6a to match the excitons of ${\text{MoS}}_{2}$ and ${\text{MoSe}}_{2}$ monolayers. As shown in Figure 6b–c, the plasmonic EQ mode is tunable by varying the Au‐cavity thickness. This tunability is a prerequisite for achieving and quantifying strong coupling in Au–${\text{MoS}}_{2}$ and Au–${\text{MoSe}}_{2}$, and it reveals clear anticrossing with Rabi splittings that persist to room temperature (see Figure 6d–e). The extracted Rabi splitting of Au–${\text{MoS}}_{2}$ heterostructures is 112.2 meV (near zero detuning), and the resulting polariton hybrid states exhibit pronounced anharmonicity, supporting high‐purity and offering a route to efficient out‐coupling.

**FIGURE 6:**
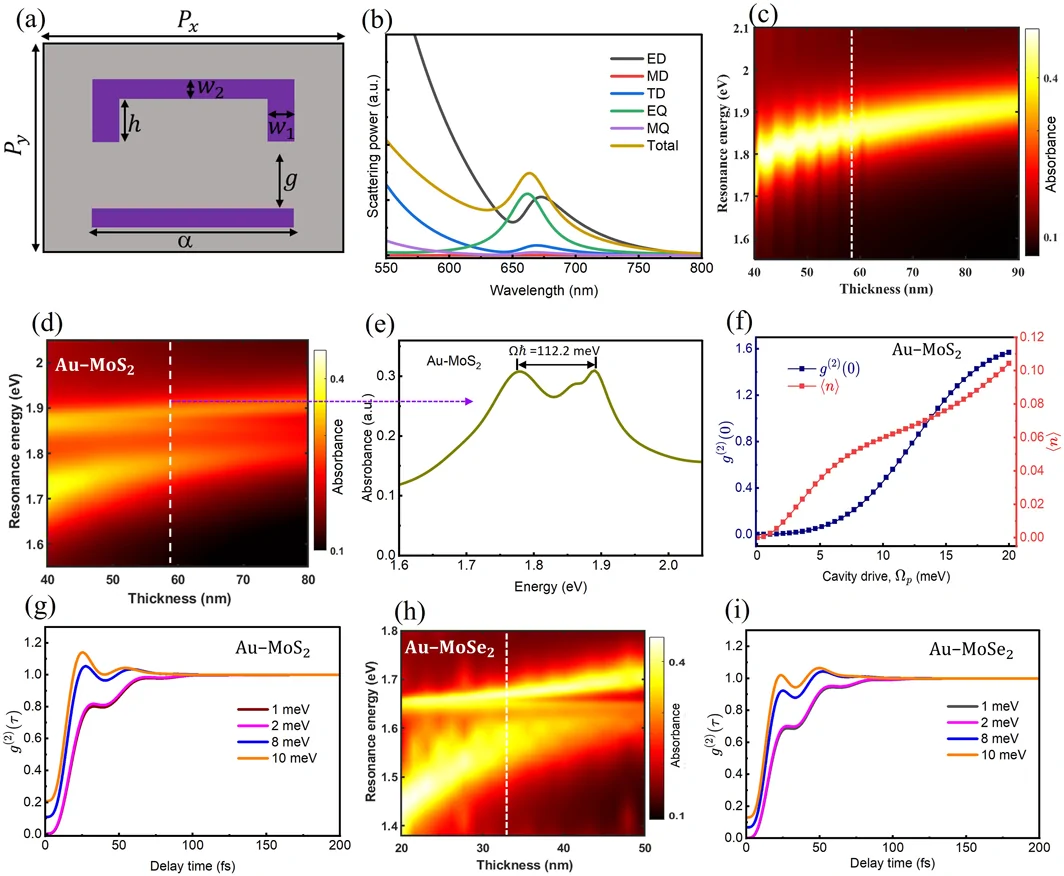
SP emission in strongly coupled Au–TMDCs through UnPB (a) Top view of a unit cell of the Au metasurface designed to match with A‐excitons of ${\text{MoS}}_{2}$ monolayer with design parameters of $W_{1}=39$ nm, $W_{2}=33$ nm, h=96 nm, g=126 nm, α=307 nm, and $P_{x}=P_{y}$ = 400 nm. (b) Multipole analysis of Au cavity at t=58.4  nm showing the dominance of EQ contribution. (c) Effect of Au thickness on plasmon resonance tuning. The vertical dashed line indicates that at a thickness of t=58.4 nm of the Au cavity, the plasmon mode absorption spectra match with the A‐exciton transition of ${\text{MoS}}_{2}$. (d) Absorption spectra of Au–${\text{MoS}}_{2}$ hybrid structures with a design parameter of Au cavity shown in panel (a) with varying Au thickness from 40 to 80 nm. (e) Absorbance of Au–${\text{MoS}}_{2}$ at detuning. The corresponding Hopfield coefficients quantifying exciton and plasmon contributions to the hybrid polariton modes of panel (d) are shown in Supporting Information S1: Figure S3. (f) Effect of drive amplitude $\Omega_{p}$ on $g^{\left(2\right)}\left(0\right)$ and mean photon number 〈n〉. (g) $g^{\left(2\right)}\left(\tau \right)$ for Au–${\text{MoS}}_{2}$ at $\Omega_{p}=1,2,8,10$  meV at room temperature. (h) Absorption mapping of Au–${\text{MoSe}}_{2}$ with varying Au thickness. (i) Calculated $g^{\left(2\right)}\left(\tau \right)$ for an Au–${\text{MoSe}}_{2}$ exciton–plasmon system at cavity‐drive amplitudes of $\Omega_{p}=1,2,8$, and 10meV. All curves exhibit a pronounced antibunching dip at τ=0.

As a result, the formation of exciton–plasmon strong coupling at room temperature in the Au–${\text{MoS}}_{2}$ system enables pronounced photon blockade: excitation of a single polariton suppresses subsequent excitation because the polaritonic ladder is anharmonic. To further enhance antibunching, the simultaneous driving of the cavity and exciton modes was optimized (see Equation 23) to achieve an UnPB condition through destructive interference between the two excitation pathways. Under this two‐tone excitation, the system yields the extremely low $g^{\left(2\right)}\left(0\right)$ values observed in Figure 6f–g. The robustness of the photon blockade in this system results in an ultra‐low $g^{\left(2\right)}\left(0\right)$ value of $2.30\times 10^{-4}$ at a cavity drive of $\Omega_{p}=0.5$ meV and a coupling strength of 56.11 meV. Such deep antibunching indicates near‐perfect SPE, representing a roughly three‐order‐of‐magnitude improvement in multiphoton suppression over typical uncoupled ${\text{MoS}}_{2}$ quantum emitters [46]. Additionally, embedding the monolayer in an Au metasurface yields large Rabi splittings and tight confinement, enabling blockade dominated by cavity anharmonicity rather than localized‐defect nonlinearity, thereby suppressing multiphoton probability even under modest drive.

Furthermore, under identical coherent driving conditions, the designed Au–${\text{MoSe}}_{2}$ hybrid structure was also investigated for SPE applications. Unlike quantum emission from monolayer ${\text{MoSe}}_{2}$ at cryogenic temperatures [19], here, we study operation at room temperature. Tuning the Au‐cavity thickness allows spectral matching to the ${\text{MoSe}}_{2}$ monolayer exciton. This system exhibits strong coupling at room temperature with a large Rabi splitting exceeding 135 meV (see Figure 6h). This Rabi splitting increases as the Au thickness decreases, consistent with enhanced field confinement and a smaller mode volume in thinner and narrower cavities [28]. Subsequently, as shown in Figure 6i the induced enhanced nonlinear response with moderate radiative damping in Au‐ ${\text{MoSe}}_{2}$, making it suitable for photon blockade under the same dual‐drive configuration. This results in an ultra‐low $g^{\left(2\right)}\left(0\right)$ value of $1.59\times 10^{-4}$ at $\Omega_{p}=0.5$ meV and a coupling strength of 67.5 meV, with purity increasing as the drive amplitude $\Omega_{p}$ is reduced.

As shown in Figure 6i, the $g^{\left(2\right)}\left(\tau \right)$ reveals how the single‐photon character evolves with delay. For all drive amplitudes $\Omega_{p}$, the correlations exhibit a deep antibunching dip at zero delay, $g^{\left(2\right)}\left(0\right)\;\ll \;1$, confirming strong suppression of simultaneous two‐photon emission due to destructive interference between the cavity‐ and exciton‐driven excitation pathways. At longer timescales, $g^{\left(2\right)}\left(\tau \right)$ rises above unity and shows a modest overshoot before relaxing to $g^{\left(2\right)}\left(\tau \right)\;\to \;1$, reflecting re‐excitation dynamics and damped Rabi oscillations of the coupled exciton–plasmon system. Increasing the drive from $\Omega_{p}=1$ to 10meV gradually fills in the antibunching dip and enhances the overshoot, indicating power broadening and an increased probability of populating higher excitation manifolds. Nevertheless, the curves retain clear antibunching at τ=0, demonstrating that UnPB remains effective over a broad range of drive strengths in Au–${\text{MoSe}}_{2}$.

Together, Au–${\text{MoS}}_{2}$ and Au–${\text{MoSe}}_{2}$ complement Au–${\text{WSe}}_{2}$ by spanning different exciton energies and dephasing environments, enabling a comparative Au‐metasurface–tunable platform in which detuning, Rabi splitting, and radiative channels can be co‐optimized to realize scalable, on‐chip SP emission with engineered $g^{\left(2\right)}\left(0\right)$ and directional out‐coupling.

### 3.5 Antibunching in Weakly Coupled Au‐${\text{WSe}}_{2}$

Finally, to validate our model for SPS purity in the strong coupling regime, we transitioned to the weak‐coupling regime by decreasing the Q‐factor and field enhancement of the Au cavity (see Figure 7a). Here, the emitter is placed in the near field of the plasmonic structure, allowing spontaneous emission to couple into plasmonic modes. The design parameters of the Au cavity were set to ($W_{1}=96$ nm, $W_{2}=90$ nm, h=86.5 nm, g=86.5 nm, α=348 nm, t=80 nm, and $P_{x}=P_{y}=420$ nm) to match the exciton resonance of ${\text{WSe}}_{2}$. The optical response of the designed metasurface is strongly dependent on the cavity gap width [28]. As shown in Figure 7b–c, the bare metasurface exhibits an electric field enhancement of ${\left|E/E_{0}\right|}^{2}=106$ and a broad, low‐Q absorption spectrum. Although the field enhancement of the Au cavity is lower than that of Figure 1c (with narrower slit gaps), it still reaches ${\left|E/E_{0}\right|}^{2}=191$ when hybridized with ${\text{WSe}}_{2}$. Despite this enhancement, distinct polariton branches are not observed due to the reduced local density of optical states (LDOS) induced by the Au cavity, which prevents hybrid state formation. The coupling strength scales as $g\propto Q/\sqrt{V}$, where Q is the cavity quality factor and V its optical mode volume. Therefore, before simulating $g^{\left(2\right)}\left(0\right)$ for the ${\text{WSe}}_{2}$ emitter coupled to the Au cavity, we first verified the coupling strength. According to Equation (15), if the calculated Rabi splitting in Au–${\text{WSe}}_{2}$ does not satisfy both criteria, the system lies in the weak‐coupling regime [41]. By substituting $\Gamma_{P}=125.9$ meV from Figure 7 into Equation (15), both conditions are unmet, confirming weak coupling. As shown in Figure 7f, this regime yields a Purcell factor $F_{P}$ that quantifies the enhancement of the ${\text{WSe}}_{2}$ emission rate $\Gamma_{X}$ through the plasmonic cavity, expressed as $\gamma =\left(1+F_{P}\right)\Gamma_{X}$, where $F_{P}\propto {\left(\lambda /n\right)}^{3}\left(Q/V\right)$, indicating that strong field confinement enhances spontaneous emission. The calculated $F_{P}$ at a 10 nm emitter–cavity separation is 1310, which exceeds the 15±3 reported for ${\text{WSe}}_{2}$ in a plasmonic slot waveguide, resulting in $g^{\left(2\right)}\left(0\right)=0.42$, consistent with weaker field confinement in our metasurface [47]. Notably, $F_{P}=1310$ is moderate compared to $F_{P}>10^{5}$ achievable when the dipole emitter is closely aligned with the local field maximum in the ${\text{WSe}}_{2}$ (see Supporting Information S1: Figure S4).

**FIGURE 7:**
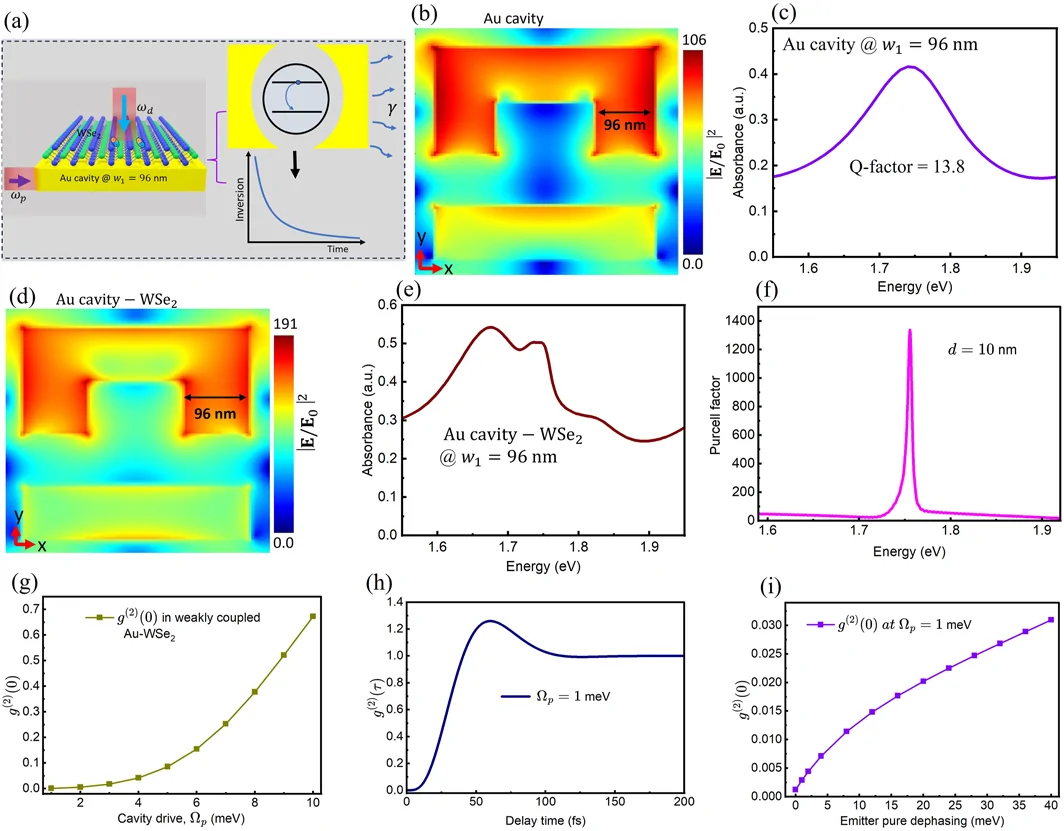
Photon antibunching in the weak coupling regime of Au–${\text{WSe}}_{2}$. (a) Schematic of a weakly coupled Au cavity with a monolayer of ${\text{WSe}}_{2}$ placed in the near field; spontaneous emission (SE) photons are funneled into radiative and plasmonic channels set by the LDOS. (b–c) Electric‐field enhancement ${\left|E/E_{0}\right|}^{2}$ and the corresponding plasmon absorption resonance of the bare Au cavity at $W_{1}=96$ nm. (d–e) Near‐field enhancement ${\left|E/E_{0}\right|}^{2}$ for the hybrid Au–${\text{WSe}}_{2}$ nanostructure and its corresponding absorption spectrum, showing exciton–plasmon interaction signatures in the weak‐coupling regime. (f) Purcell factor $\left(F_{P}\right)$ extracted for an in‐plane ${\text{WSe}}_{2}$ dipole positioned 10 nm from the hybrid structure. (g) Effect of cavity drive on $g^{\left(2\right)}\left(0\right)$, which increases with drive as multiphoton occupation becomes more probable, degrading antibunching. (h) $g^{\left(2\right)}\left(\tau \right)$ at $\Omega_{p}=1\;meV$ showing an antibunching dip at $\tau =0\;\left(g^{\left(2\right)}\left(0\right)<1\right)$ and relaxation toward $g^{\left(2\right)}\left(\infty \right)=1$ at long delays. (i) Dependence of $g^{\left(2\right)}\left(0\right)$ on the emitter pure‐dephasing rate (in meV).

Subsequently, we computed the time evolution of the ${\text{WSe}}_{2}$ emitter coupled to the wider‐gap Au cavity shown in Figure 7g, operating in the UnPB scheme. In this configuration, quantum interference between two excitation pathways is exploited to suppress multiphoton occupation even in parameter regimes where CPB would fail. By coherently driving both the cavity and the ${\text{WSe}}_{2}$ emitter with amplitudes related by the analytical condition in Equation (23), the destructive interference cancels the two‐photon component of the intracavity field [35]. This results in a pronounced reduction of multiphoton probability and a highly sub‐Poissonian field with $g^{\left(2\right)}\left(0\right)=0.0012$. The degree of photon antibunching and the source purity were further quantified through the time‐dependent correlation $g^{\left(2\right)}\left(\tau \right)$. Figure 7h, computed using Equation (17), shows a deep antibunching dip at τ=0 followed by relaxation toward $g^{\left(2\right)}\left(\tau \right)\to 1$, demonstrating strong suppression of multiphoton events in the weak‐coupling regime. Although the antibunching is slightly less pronounced than in the strong coupling case shown in Figure 5, the UnPB mechanism remains highly effective and underscores the potential of quantum nanoplasmonics to realize high‐purity SPSs beyond the conventional strong coupling regime.

Although our simulations are performed at room temperature, real devices may exhibit additional linewidth broadening due to enhanced exciton–phonon coupling, which increases the pure dephasing rate $\Gamma_{\phi }$ [48]. Therefore, to account for this effect, the master equation in Equation (7) is modified by including an additional emitter pure‐dephasing term, as follows:

(24) $$\dot{\rho }=-i\;\left[H,\rho \right]+D\left[\sqrt{\Gamma_{P}}\;a\right]\rho +D\left[\sqrt{\Gamma_{X}}\;\sigma^{-}\right]\rho +D\left[\sqrt{\Gamma_{\phi }}\;\sigma^{+}\sigma^{-}\right]\rho .$$

This modified master‐equation model is used to evaluate the robustness of photon antibunching under pure dephasing, as shown in Figure 7i. Pure dephasing suppresses the phase coherence between the ground and excited states of the ${\text{WSe}}_{2}$ emitter without altering the level populations, thereby converting a coherent superposition into an incoherent mixture. Nevertheless, under hybrid coupling to the Au metasurface, our system still maintains strong antibunching, with $g^{\left(2\right)}\left(0\right)=0.030$ even when the pure dephasing is increased up to 40 meV at room temperature, as shown in Figure 7i.

### 3.6 Effect of Dielectric Spacer on Photon Purity in TMDCs/Au Metasurface/${SiO}_{2}$

To further assess the experimental feasibility of the proposed device, we rigorously investigated the influence of the spacer within the numerical simulations. Because the present work is primarily based on simulations and theoretical modeling, these additional calculations were performed to bring the design closer to realistic fabrication conditions. In the proposed structure, a thin alumina (${\text{Al}}_{2}O_{3}$) dielectric spacer with a refractive index of n=1.6 is introduced between the ${\text{MoSe}}_{2}$ and ${\text{WSe}}_{2}$ monolayers to prevent direct metal‐induced quenching (see Supporting Information S1: Figures S5 and S6 in the supplementary information). As a result, first the design parameters of the Au metasurface shown in Supporting Information S1: Figure S5a were varied to match the exciton of ${\text{MoSe}}_{2}$. As shown in Supporting Information S1: Figure S5c, the plasmon resonance of the Au metasurface/SiO2 matches the ${\text{MoSe}}_{2}$ resonances at a thickness of t=54 nm. Subsequently, ${\text{Al}}_{2}O_{3}$ varied from 2 to 3 nm in 1L‐${\text{MoSe}}_{2}$/${Al}_{2}O_{3}$/Au metasurface/${SiO}_{2}$ substrate, as shown in Supporting Information S1: Figure S5d [49]. In addition, we evaluated the influence of the ${\text{Al}}_{2}O_{3}$ spacer on the Rabi splitting. As shown in Supporting Information S1: Figures S5e and S6b in the supplementary information, the Rabi splitting is only weakly affected by the presence of the dielectric spacer, confirming that a few‐nanometer‐thick ${\text{Al}}_{2}O_{3}$ layer can effectively suppress metal‐induced quenching without significantly degrading the coupling strength. Using the extracted plasmon linewidth $\kappa_{P}$, exciton linewidth $\Gamma_{X}$, and coupling strength estimated from the Rabi splitting in Supporting Information S1: Figure S5e, the calculated $g^{\left(2\right)}\left(0\right)$ exhibits strong antibunching at a drive strength of 1 meV (see Supporting Information S1: Figure S5f in the supplementary information). Furthermore, to account for defect‐induced decoherence that may arise during ${\text{MoSe}}_{2}$ monolayer transfer, we introduced a pure‐dephasing term $\Gamma_{\phi }$ into Equation (24). As shown in Supporting Information S1: Figure S5f, $g^{\left(2\right)}\left(0\right)$ remains as low as 0.02 even in the presence of pure dephasing, corresponding to a high single‐photon purity. Additionally, these results confirm that the proposed 1L‐${\text{WSe}}_{2}$/spacer/Au metasurface platform shown in Supporting Information S1: Figure S6 can also retain high‐purity antibunched emission under realistic experimental conditions. Moreover, the designed Au metasurfaces, shown in Figure 7b and Supporting Information S1: Figure S5b in the supplementary information, exhibits strong field confinement and forms deterministic plasmonic hot spots across the array. When a TMDC monolayer is transferred onto these structures, strain‐localized emitter states positioned near these gaps can efficiently couple to the enhanced local optical field. As a result, this could enable cavity‐assisted deterministic SPE with enhanced both the radiative emission rate and quantum yield [22].

Furthermore, the proposed design fabrication process can be carried out using conventional electron‐beam lithography (EBL) and lift‐off techniques. First, a resist layer can be patterned on the ${\text{SiO}}_{2}$ substrate by EBL, followed by development and surface cleaning. A thin Ti adhesion layer is then deposited by electron‐beam evaporation under high‐vacuum conditions, followed by deposition of the Au layer. After lift‐off, a thin ${\text{Al}}_{2}O_{3}$ dielectric spacer of 2–3 nm is deposited by atomic layer deposition (ALD). Alternatively, a thin hexagonal boron nitride (hBN) flake may be transferred as an atomically flat dielectric spacer to prevent direct contact between the TMDC monolayers and the Au metasurface. Finally, a crystalline ${\text{WSe}}_{2}$ (including other TMDC) monolayers can be transferred onto the spacer‐coated Au metasurface to evaluate the resulting strong coupling and SPE performance.

## 4 Conclusions

Solid‐state single‐photon sources that operate at room temperature and are compatible with planar nanophotonic integration remain a key unmet need for scalable quantum photonic technologies. In this study, we theoretically demonstrate room‐temperature SPE in TMDC monolayers by strongly coupling them to an Au plasmonic metasurface. First, the Au metasurface was characterized through multipole decomposition, and strong coupling was established by fitting a coupled‐oscillator model to the polariton dispersion, which reproduces the pronounced anticrossing and yields vacuum Rabi splittings exceeding 100 meV at room temperature. This coupling rate is enabled by the metasurface's high‐Q EQ mode and deep‐subwavelength mode confinement, which enhances Q/V and thus exciton–plasmon coupling strength g. Across the three hybrids considered here, Au–${\text{WSe}}_{2}$, Au–${\text{MoS}}_{2}$, and Au–${\text{MoSe}}_{2}$, we obtain coupling strengths g in the tens of meV, corresponding to high cooperativities in Au–TMDC heterostructures. This quantum nanoplasmonic design, which engineers intense, localized near fields at exciton resonances, enables robust strong coupling and single‐photon operation at room temperature despite substantial dephasing in TMDC monolayers. Building on this strong‐coupling platform, we simulated deterministic single‐photon generation using both CPB (single‐drive) and UnPB (interference‐assisted, two‐drive) within a JCs master‐equation framework. For Au–${\text{WSe}}_{2}$ in the CPB regime, resonant driving at the lower polariton yields deep antibunching with $g^{\left(2\right)}\left(0\right)\;\approx \;1.4\times 10^{-3}$ at $\Omega_{p}=0.002$ meV, and even lower values approaching $10^{-4}$ under optimal weak‐drive conditions. In the UnPB configuration, the two‐drive scheme further suppresses multiphoton events, as confirmed by higher‐order correlations $g^{\left(n\right)}\left(0\right)$ with n=3–5, which remain ≪1 over a broad range of $\left(g,\Omega_{p}\right)$. Extending this strategy to Au–${\text{MoS}}_{2}$ and Au–${\text{MoSe}}_{2}$ under the same dual‐drive protocol, we obtain ultra‐low of $g^{\left(2\right)}\left(0\right)=2.30\times 10^{-4}$ at $\Omega_{p}=0.5$ meV and g=56.11 meV for Au–${\text{MoS}}_{2}$, and $g^{\left(2\right)}\left(0\right)=1.59\times 10^{-4}$ at $\Omega_{p}=0.5$ meV and g=67.5 meV for Au–${\text{MoSe}}_{2}$. These values correspond to single‐photon purities $1-g^{\left(2\right)}\left(0\right)$ exceeding 99.98% at room temperature. Thus, the strongly coupled Au–TMDC heterostructures yield a two‐to three‐orders of magnitude reduction in $g^{\left(2\right)}\left(0\right)$ at room temperature compared with weak coupling operation, indicating substantial suppression of multiphoton contributions. Importantly, the high purity is maintained at non‐negligible intracavity populations: for comparable 〈n〉, the antibunching dip in $g^{\left(2\right)}\left(0\right)$ remains pronounced. Taken together, Au–${\text{WSe}}_{2}$, Au–${\text{MoS}}_{2}$, and Au–${\text{MoSe}}_{2}$ form a tunable quantum nanophotonic platform in which exciton energy, coupling strength, dephasing environment, and drive configuration can be co‐engineered to realize scalable on‐chip SP emitters with scalable $g^{\left(2\right)}\left(0\right)$ and directional out‐coupling. In this way, our room‐temperature Au metasurface–TMDC platform could overcome the traditional reliance on cryogenic TMDC monolayer SPSs and establish a viable route toward integrated quantum light sources for quantum key distribution and low‐power photonic quantum interconnects.

## Author Contributions

**Andergachew Mekonnen Berhe:** conceptualization, investigation, writing – original draft, methodology, software, formal analysis, project administration, visualization, data curation. **Haroldo T. Hattori:** writing – review and editing, supervision. **Andrey E. Miroshnichenko:** funding acquisition, writing – review and editing, software, supervision, methodology.

## Conflicts of Interest

The authors declare no conflicts of interest.

## Supporting information


Supporting Information S1

## Data Availability

The data that support the findings of this study are available from the corresponding author upon reasonable request.
