# Supplementary material for: Single‐Photon Emission From TMDCs through Strong Coupling to EQ Plasmon Modes of an Au Metasurface
Source: Nanophotonics. 2026 May 29;15(11):e70147. doi: 10.1002/nap2.70147 (PMC13642101; doi:10.1002/nap2.70147)
Supplement: Supplementary file 1 [file NAP2-15-e70147-s001.pdf]

Supplementary Information

Single-Photon Emission from TMDCs through Strong Coupling to EQ Plasmon Modes of an Au Metasurface

*Andergachew Mekonnen Berhe, Haroldo T. Hattori, and Andrey E. Miroshnichenko**

A. M. Berhe, H. T. Hattori, and A. E. Miroshnichenko
Address: School of Engineering and Technology, University of New South Wales at Canberra, Northcott Drive, Canberra ACT 2610, Australia
Email Address: andrey.miroshnichenko@unsw.edu.au

S1: Multipole decomposition within an Au metasurface

The multipole moments are calculated using FDTD and defined as:[1]

$$\mathbf{P} = \frac{1}{i\omega}\int \mathbf{j}\, d^3r, \tag{1}$$

$$\mathbf{M} = \frac{1}{2c}\int (\mathbf{r}\times\mathbf{j})\, d^3r, \tag{2}$$

$$\mathbf{T} = \frac{1}{10c}\int \left[(\mathbf{r}\cdot\mathbf{j})\,\mathbf{r} - 2r^2\mathbf{j}\right] d^3r, \tag{3}$$

$$Q^{(e)}_{\alpha\beta} = \frac{1}{2i\omega}\int \left[r_\alpha j_\beta + r_\beta j_\alpha - \frac{2}{3}(\mathbf{r}\cdot\mathbf{j})\,\delta_{\alpha\beta}\right] d^3r, \tag{4}$$

$$Q^{(m)}_{\alpha\beta} = \frac{1}{3c}\int \left[(\mathbf{r}\times\mathbf{j})_\alpha r_\beta + (\mathbf{r}\times\mathbf{j})_\beta r_\alpha\right] d^3r\ . \tag{5}$$

where c is the speed of light, ω is the angular frequency, and $\alpha, \beta = x, y, z$. The multipoles $\mathbf{P}$, $\mathbf{M}$, $\mathbf{T}$, Q^e, and Q^m denote electric dipole, magnetic dipole, toroidal dipole, electric quadrupole, and magnetic quadrupole moments, respectively. The scattered powers of these multipoles are calculated using:

$$I_P = \frac{2\omega^4}{3c^3}|\mathbf{P}|^2, \tag{6}$$

$$I_M = \frac{2\omega^4}{3c^3}|\mathbf{M}|^2, \tag{7}$$

$$I_T = \frac{2\omega^6}{3c^5}|\mathbf{T}|^2, \tag{8}$$

$$I_{Q^{(e)}} = \frac{\omega^6}{5c^5}\sum_{\alpha,\beta}|Q^{(e)}_{\alpha\beta}|^2, \tag{9}$$

$$I_{Q^{(m)}} = \frac{\omega^6}{40c^5}\sum_{\alpha,\beta}|Q^{(m)}_{\alpha\beta}|^2\ . \tag{10}$$

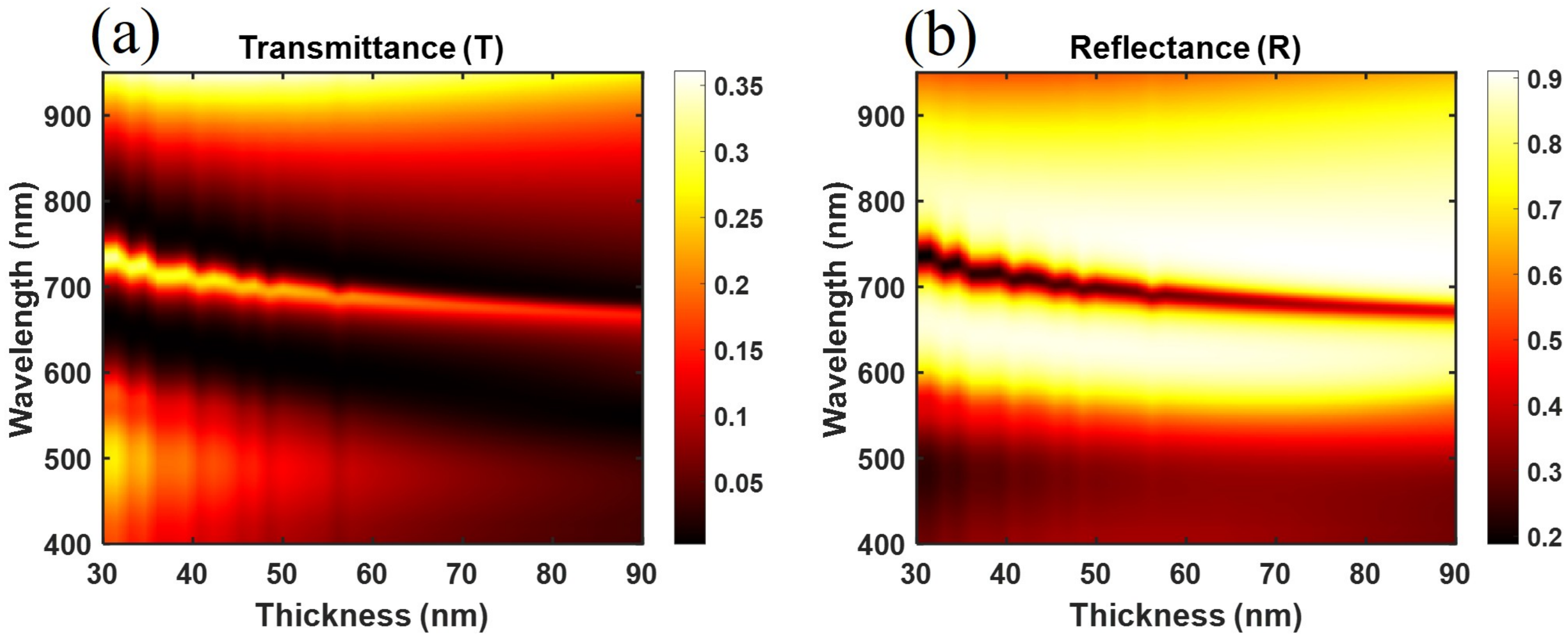

Figure S1: The corresponding sweep of transmittance (a) and reflectance (b) for Figure 2(b). These results show that varying Au thickness induces tunable optical properties that can be matched to the excisions of TMDCs.

Subsequently, the total scattered power is then calculated from the following formula:

$$I = \frac{2\omega^4}{3c^3}|\mathbf{P}|^2 + \frac{2\omega^4}{3c^3}|\mathbf{M}|^2 + \frac{4\omega^5}{3c^4}(\mathbf{P}\cdot\mathbf{T}) + \frac{2\omega^6}{3c^5}|\mathbf{T}|^2 + \frac{\omega^6}{5c^5}\sum_{\alpha,\beta}|Q^{(e)}_{\alpha\beta}|^2 + \frac{\omega^6}{40c^5}\sum_{\alpha,\beta}|Q^{(m)}_{\alpha\beta}|^2 + O\left(\frac{1}{c^5}\right). \tag{11}$$

Here, we integrate the light-induced polarization over a single element of the infinite periodic Au metasurface to calculate the multipole contributions to the scattered electric-field amplitude. As a result, in our multipole decomposition approach, we consider the electric dipole (ED) moment $\mathbf{P}$, the magnetic dipole (MD) moment $\mathbf{M}$, the electric quadrupole (EQ) tensor $\hat{Q}^{(e)}$, the magnetic quadrupole (MQ) tensor $\hat{Q}^{(m)}$, and the toroidal dipole moment $\mathbf{T}$ (see Fig. 1 in the main text).

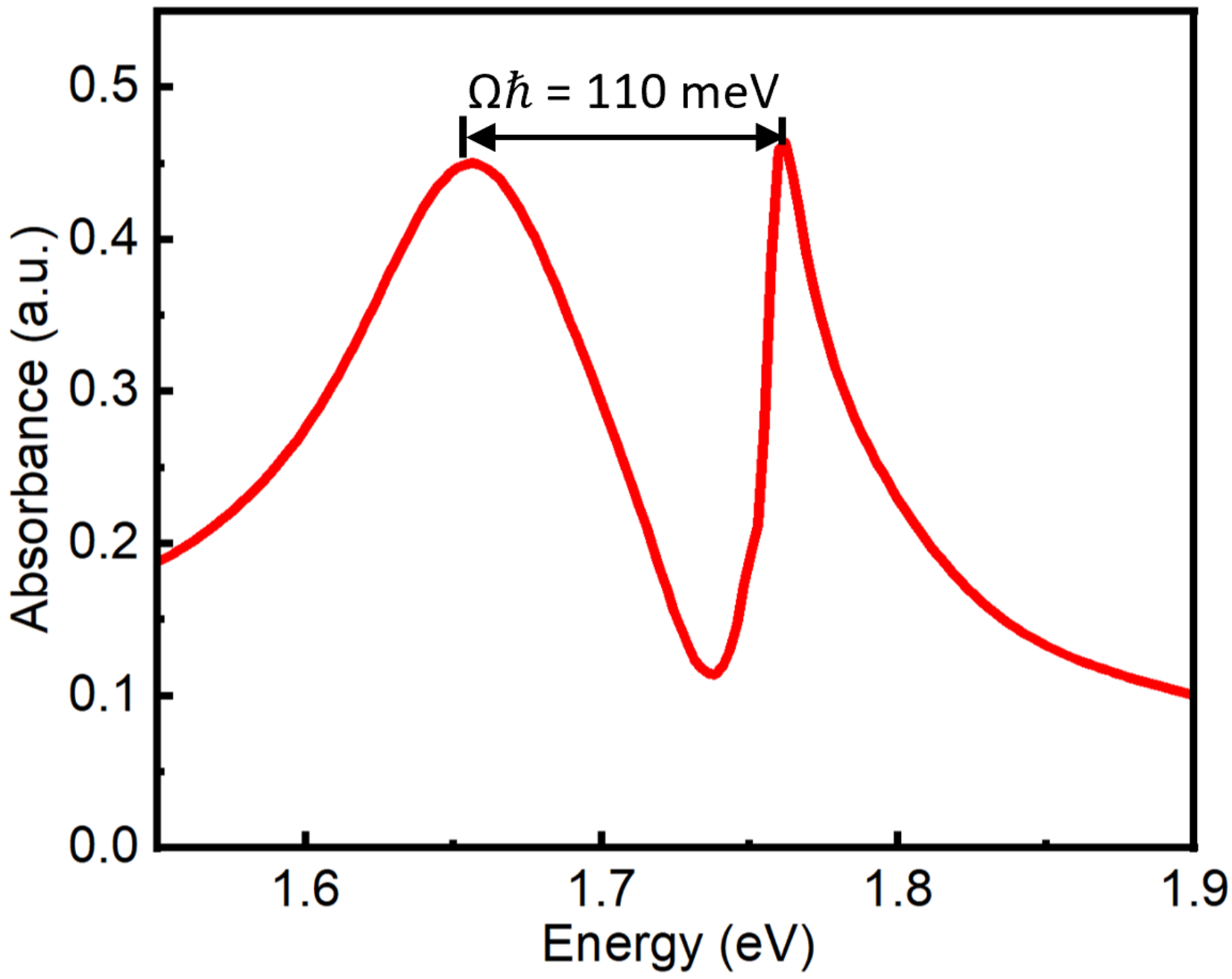

Figure S2: Polariton branches of Au-WSe_2 nanostructures induced at a thickness of 42.3 nm, at which the uncoupled Au metasurface matches with the exciton of WSe_2. See Fig. 3(f) for the effect of Au thickness on Rabi splitting as well as the bare plasmon modes in Figs. 2(d-e) in the main text.

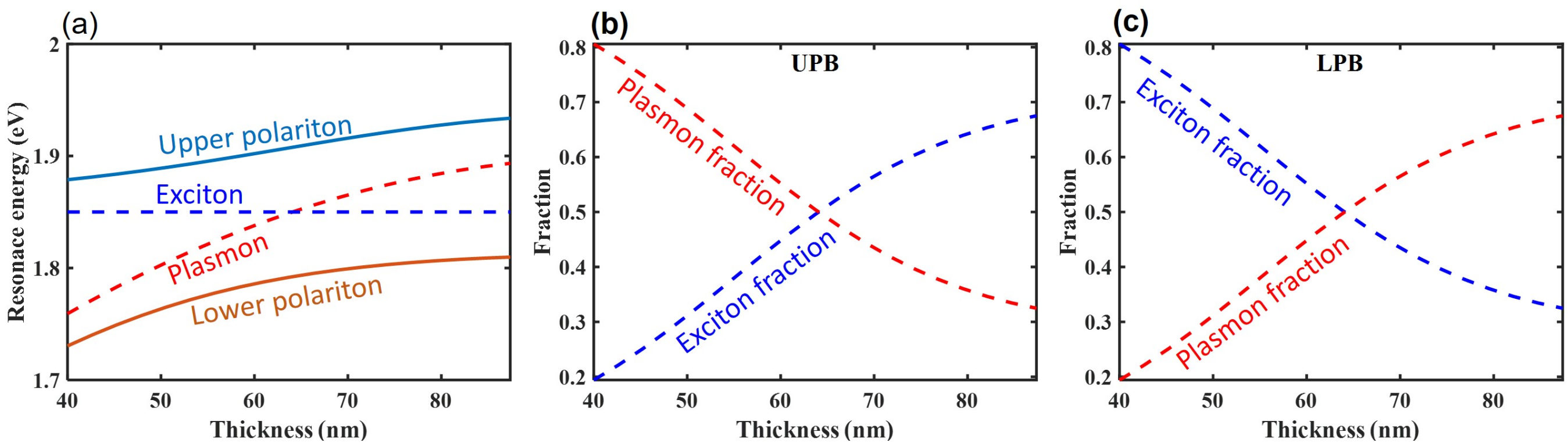

Figure S3: Dispersion relation of Au-MoS_2 of Fig. 6(d) in the main text. (a) Dispersion curve of Au–MoS_2 heterostructures as a function of thickness, where the solid lines and dashed lines represent the upper polariton branch (UPB), lower polariton branch (LPB), uncoupled plasmonic resonances, and uncoupled MoS_2 exciton resonance, respectively. (b-c) The corresponding Hopfield coefficients for MoS_2 exciton and Au plasmonic contributions to UPB and LPB as a function of thickness.

S2: Anharmonicity of the JCs Ladder and the Two–Photon Blockade Shift in SC

In the resonant JCs model, where the cavity and emitter frequencies satisfy $\omega_c = \omega_x$, the eigenstates form a ladder of dressed polaritonic doublets. In the n-excitation manifold, the corresponding eigenenergies are

$$E_{n,\pm} = n\hbar\omega_c \;\pm\; \hbar g\sqrt{n}, \tag{12}$$

highlighting the characteristic $\sqrt{n}$ nonlinearity responsible for the anharmonic structure of the JC ladder. For the first excited manifold ($n = 1$), the lower and upper polaritons lie at

$$E_{1,-} = \hbar(\omega_c - g), \qquad E_{1,+} = \hbar(\omega_c + g), \tag{13}$$

while the second-excitation manifold ($n = 2$) contains the dressed states

$$E_{2,-} = 2\hbar\omega_c - \hbar g\sqrt{2}, \qquad E_{2,+} = 2\hbar\omega_c + \hbar g\sqrt{2}. \tag{14}$$

To quantify the anharmonicity relevant for photon blockade, we compare the second-rung energies with the values one would obtain if the ladder were strictly harmonic. For the lower polariton (LP) branch, the harmonic expectation is $2E_{1,-}$, whereas the true JC energy is $E_{2,-}$. The difference,

$$\begin{aligned} \Delta_{\text{blk}} &= E_{2,-} - 2E_{1,-} \\ &= \big(2\hbar\omega_c - \hbar g\sqrt{2}\big) - 2\big(\hbar\omega_c - \hbar g\big) \\ &= (2 - \sqrt{2})\,\hbar g, \end{aligned} \tag{15}$$

defines the intrinsic JC blockade shift.

$$\boxed{\Delta_{\text{blk}} = (2 - \sqrt{2})g}.$$

This anharmonicity underpins CPB. When the cavity is driven near the lower-polariton frequency, $\omega_p \approx \omega_c - g$, the single-photon transition $|0, g\rangle \to |1, -\rangle$ is resonant, while the subsequent two-photon step $|1, -\rangle \to |2, -\rangle$ is detuned by Δ_{blk} (See Fig. 4(a) in the main text).

S3: Derivation of the two-photon interference (UnPB) condition in Au–WSe_2 TMDCs, Eq. 23 in the main text

This section derives the analytic "two-photon cancellation" condition used in the main text. Working in the weak-driving steady state and truncating the Hilbert space to at most two total excitations [Eq. (20) in the main text], we obtain a closed set of equations for the amplitudes $\{c_{0g}, c_{1g}, c_{0e}, c_{2g}, c_{1e}\}$. The key quantity is the two-photon amplitude c_{2g} associated with $|2, g\rangle$; enforcing $c_{2g} = 0$.

S3.1 Weak-driving truncation and effective non-Hermitian dynamics

In the weak-driving regime, the system remains close to the ground state and higher excitation manifolds are negligibly populated. We therefore approximate the system state as [2]

$$|\psi(t)\rangle \approx c_{0g}|0, g\rangle + c_{1g}|1, g\rangle + c_{0e}|0, e\rangle + c_{2g}|2, g\rangle + c_{1e}|1, e\rangle + \cdots, \tag{16}$$

dropping states with three or more total excitations. Dissipation is incorporated through a non-Hermitian Schrödinger equation

$$i\partial_t|\psi\rangle = H_{\rm nH}|\psi\rangle, \tag{17}$$

with the same effective Hamiltonian used in the main text. In the rotating frame, we write

$$H_{\rm nH} = \tilde{\Delta}_c\, a^\dagger a + \tilde{\Delta}_x\, \sigma_+\sigma_- + g\,(a\sigma_+ + a^\dagger\sigma_-) + \left(\Omega_p\, a^\dagger + \Omega_p^*\, a\right) + \Omega_d\, \sigma_+ + \Omega_d^*\, \sigma_-, \tag{18}$$

where $\tilde{\Delta}_c$ and $\tilde{\Delta}_x$ are complex detunings (including loss) and g is the coherent coupling strength between the plasmonic cavity and WSe_2 exciton. Throughout we use the main-text definition

$$\tilde{\Delta} \equiv \tilde{\Delta}_c + \tilde{\Delta}_x, \tag{19}$$

and, for compactness in the quadratic solution below, we define

$$\tilde{\Delta}_r \equiv \tilde{\Delta}_c. \tag{20}$$

S3.2 Projected steady-state equations in the two-excitation manifold

Projecting $i\partial_t|\psi\rangle = H_{\rm nH}|\psi\rangle$ onto the truncated basis $\{|0, g\rangle, |1, g\rangle, |0, e\rangle, |2, g\rangle, |1, e\rangle\}$ yields the coupled amplitude equations:

$$i\dot{c}_{0g} = \Omega_p^*\, c_{1g} + \Omega_d^*\, c_{0e}, \tag{21}$$
$$i\dot{c}_{1g} = \tilde{\Delta}_c\, c_{1g} + g\, c_{0e} + \Omega_p\, c_{0g} + \sqrt{2}\,\Omega_p^*\, c_{2g} + \Omega_d^*\, c_{1e}, \tag{22}$$
$$i\dot{c}_{0e} = \tilde{\Delta}_x\, c_{0e} + g\, c_{1g} + \Omega_d\, c_{0g} + \Omega_p^*\, c_{1e}, \tag{23}$$
$$i\dot{c}_{2g} = 2\tilde{\Delta}_c\, c_{2g} + \sqrt{2}\,\Omega_p\, c_{1g} + \sqrt{2}\, g\, c_{1e}, \tag{24}$$
$$i\dot{c}_{1e} = \tilde{\Delta}\, c_{1e} + \Omega_p\, c_{0e} + \Omega_d\, c_{1g} + \sqrt{2}\, g\, c_{2g}. \tag{25}$$

We now focus on the steady state ($\dot{c}_j = 0$). Under weak driving one has $c_{0g} \simeq 1$, while $c_{1g}, c_{0e} = O(\Omega)$ and $c_{2g}, c_{1e} = O(\Omega^2)$ (with Ω a typical drive scale).

S3.3 Leading order: solving for c_{1g} and c_{0e}

To first order in the drives, we neglect c_{2g} and c_{1e} in Eqs. (22)–(23) and set $c_{0g} \simeq 1$. This gives the linear system

$$0 = \tilde{\Delta}_c\, c_{1g} + g\, c_{0e} + \Omega_p, \tag{26}$$
$$0 = \tilde{\Delta}_x\, c_{0e} + g\, c_{1g} + \Omega_d. \tag{27}$$

Solving Eqs. (26)–(27) yields

$$c_{1g} = \frac{g\, \Omega_d - \tilde{\Delta}_x\, \Omega_p}{\tilde{\Delta}_c \tilde{\Delta}_x - g^2}, \tag{28}$$
$$c_{0e} = \frac{g\, \Omega_p - \tilde{\Delta}_c\, \Omega_d}{\tilde{\Delta}_c \tilde{\Delta}_x - g^2}. \tag{29}$$

S3.4 Next order and the two-photon cancellation condition $c_{2g} = 0$

We next retain c_{2g} and c_{1e} using the steady-state forms of Eqs. (24)–(25):

$$0 = 2\tilde{\Delta}_c\, c_{2g} + \sqrt{2}\, \Omega_p\, c_{1g} + \sqrt{2}\, g\, c_{1e}, \tag{30}$$
$$0 = \tilde{\Delta}\, c_{1e} + \Omega_p\, c_{0e} + \Omega_d\, c_{1g} + \sqrt{2}\, g\, c_{2g}. \tag{31}$$

From Eq. (30) we express c_{1e} in terms of c_{2g} and c_{1g}:

$$c_{1e} = -\frac{\Omega_p}{g}\, c_{1g} - \frac{\sqrt{2}\, \tilde{\Delta}_c}{g}\, c_{2g}. \tag{32}$$

Substituting Eq. (32) into Eq. (31) and then inserting the leading-order expressions (28)–(29) gives c_{2g} as a rational function of the parameters. Crucially, the condition for complete destructive interference in the two-photon manifold is simply $c_{2g} = 0$. Setting $c_{2g} = 0$ in Eq. (30) yields

$$c_{2g} = 0 \quad \Rightarrow \quad g\, c_{1e} + \Omega_p\, c_{1g} = 0 \quad \Rightarrow \quad c_{1e} = -\frac{\Omega_p}{g}\, c_{1g}. \tag{33}$$

Using Eq. (33) in the steady-state equation (25) with $c_{2g} = 0$ gives

$$0 = \tilde{\Delta}\, c_{1e} + \Omega_p\, c_{0e} + \Omega_d\, c_{1g} = -\tilde{\Delta}\, \frac{\Omega_p}{g}\, c_{1g} + \Omega_p\, c_{0e} + \Omega_d\, c_{1g}. \tag{34}$$

Substituting the leading-order solutions (28)–(29) into Eq. (34) and simplifying yields the quadratic condition quoted in the main text (see Eq. 21):

$$g^2\, \Omega_d^2 \;-\; 2g\, \tilde{\Delta}_c\, \Omega_p \Omega_d \;+\; \Omega_p^2 \big(\tilde{\Delta}_c \tilde{\Delta}_x + g^2\big) \;\simeq\; 0, \tag{35}$$

S3.5 Solving the quadratic and the square-root branch convention

Solving Eq. (35) for Ω_d yields two branches,

$$\Omega_d^{(\pm)} = \frac{\Omega_p}{g} \left(\tilde{\Delta} \;\pm\; \sqrt{\tilde{\Delta}\, \tilde{\Delta}_r - g^2} \right). \tag{36}$$

Here, since $\tilde{\Delta}$ and $\tilde{\Delta}_r$ are complex in general, the square root is multi-valued. We therefore fix the overall phase convention by choosing Ω_p real without loss of generality; with this convention, the square-root branch can be written explicitly as

$$\Omega_d^{(\pm)} = \frac{\Omega_p}{g}\left(\tilde{\Delta} \;\pm\; \mathrm{sgn}(\Omega_p)\sqrt{\tilde{\Delta}\,\tilde{\Delta}_r - g^2}\right). \tag{37}$$

S4: Derivation of $\sqrt{2}\,\Omega_p$ in the $|1,g\rangle \to |2,g\rangle$ transition (Fig. 5(a), main text)

In the Hamiltonian, we describe the coherent cavity drive by

$$H_{\mathrm{drive}} = \Omega_p\, a^\dagger + \Omega_p^*\, a, \tag{38}$$

where $\Omega_p \in \mathbb{C}$ is a single complex drive amplitude, and a, $a^\dagger$ are the annihilation and creation operators of the resonator mode. On the level diagram, this drive couples the states

$$|0,g\rangle \leftrightarrow |1,g\rangle \quad \text{and} \quad |1,g\rangle \leftrightarrow |2,g\rangle\,.$$

The crucial point here is that the *matrix elements* of H_{drive} between different photon-number states are not all equal. The difference comes entirely from the bosonic ladder-operator relations

$$a^\dagger\,|n\rangle = \sqrt{n+1}\;|n+1\rangle\,, \qquad a\,|n\rangle = \sqrt{n}\;|n-1\rangle\,. \tag{39}$$

Transition $|0,g\rangle \to |1,g\rangle$. The upward transition from $|0,g\rangle$ to $|1,g\rangle$ is driven by $a^\dagger$:

$$\begin{aligned}\langle 1,g|H_{\mathrm{drive}}|0,g\rangle &= \Omega_p\;\langle 1|a^\dagger|0\rangle\;\langle g|g\rangle && (40)\\ &= \Omega_p \cdot 1 \cdot 1 && (41)\\ &= \Omega_p. && (42)\end{aligned}$$

Thus, the effective Rabi frequency for the $|0,g\rangle \leftrightarrow |1,g\rangle$ transition is simply Ω_p, and it is natural to label this arrow in the schematic by Ω_p (see Fig 5(a) in the main text.

Transition $|1,g\rangle \to |2,g\rangle$. For the next rung of the ladder, the same drive term gives

$$\begin{aligned}\langle 2,g|H_{\mathrm{drive}}|1,g\rangle &= \Omega_p\;\langle 2|a^\dagger|1\rangle\;\langle g|g\rangle && (43)\\ &= \Omega_p\,\sqrt{1+1}\cdot 1 && (44)\\ &= \sqrt{2}\,\Omega_p. && (45)\end{aligned}$$

Here, the only difference is that the ladder operator connects $|1\rangle$ to $|2\rangle$ with a factor $\sqrt{2}$ instead of 1:

$$a^\dagger\,|0\rangle = |1\rangle\,, \qquad a^\dagger\,|1\rangle = \sqrt{2}\;|2\rangle\,. \tag{46}$$

This $\sqrt{2}$ factor is the standard bosonic enhancement for adding a photon to an already occupied cavity mode. As a result, we want the diagram to reflect the actual matrix-element strengths; the two arrows on the right-hand side should therefore be labelled as

$$|0,g\rangle \xrightarrow{\;\Omega_p\;} |1,g\rangle\,, \qquad |1,g\rangle \xrightarrow{\;\sqrt{2}\,\Omega_p\;} |2,g\rangle\,. \tag{47}$$

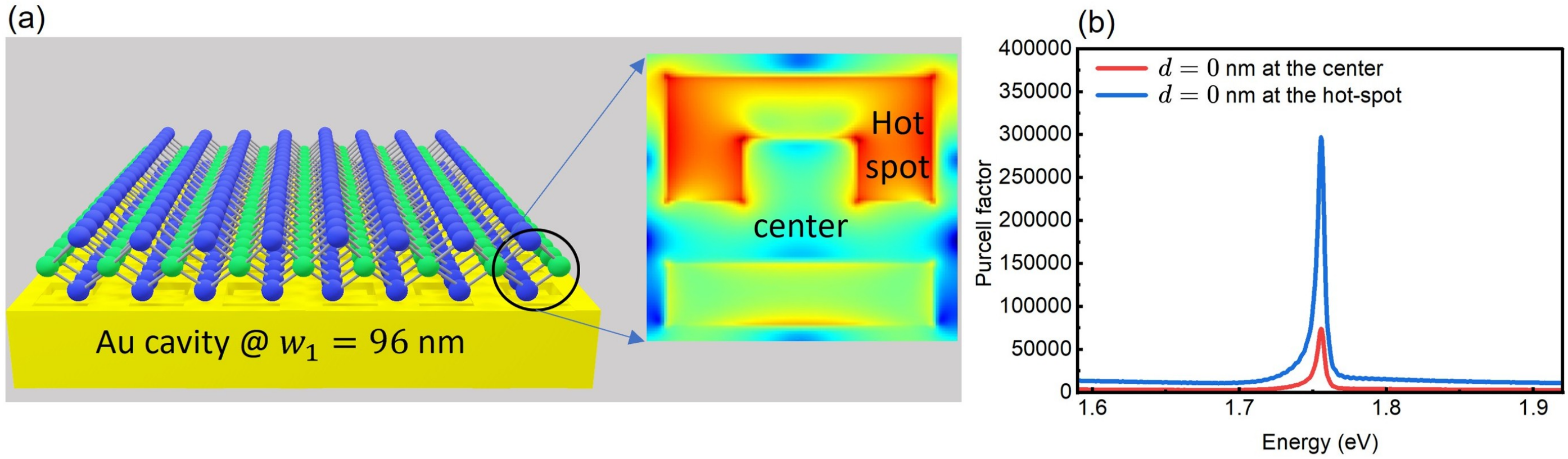

Figure S4: The schematic drawing illustrates the positions of the electric dipole emitter embedded in the weakly coupled Au-WSe_2 when the dipole emitter is placed at the WSe_2 emitter at the center (x = 0 nm and y = 0 nm). (b) The corresponding Purcell factor induced in the hybrid structure.

In words, the drive itself has the same amplitude Ω_p everywhere, but the cavity responds more strongly when there is already one photon present. That enhancement is exactly the familiar $\sqrt{n+1}$ factor from the bosonic creation operator, which for $n = 1$ becomes the $\sqrt{2}$ multiplying Ω_p on the $|1, g\rangle \to |2, g\rangle$ arrow in the schematic.

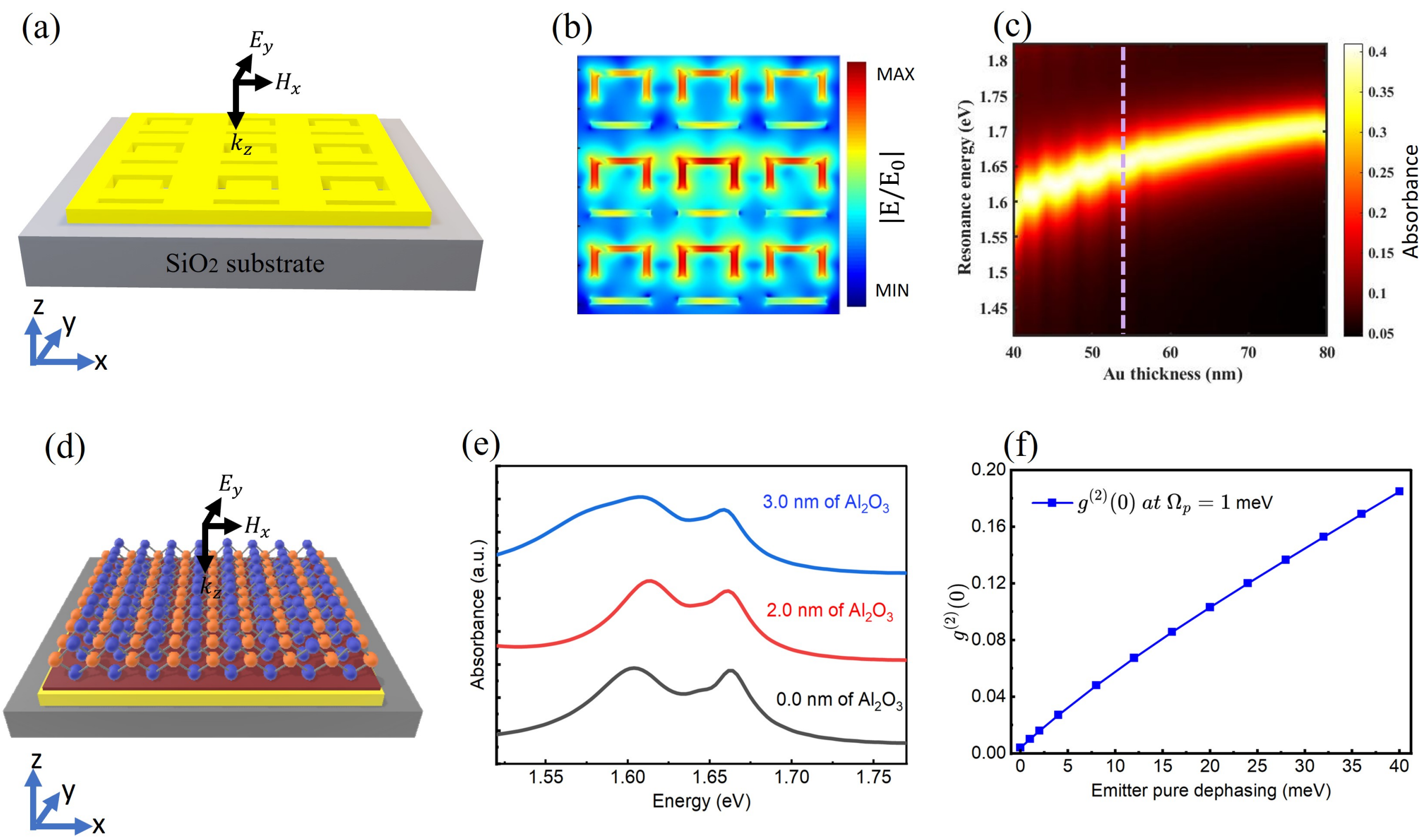

Figure S5: Effect of dielectric spacers on photon purity in a $MoSe_2$ monolayer/alumina/Au metasurface/SiO_2 heterostructure. (a) Schematic of the Au metasurface on SiO_2. (b) Corresponding electric-field profile over multiple unit cells, confirming the suitability of the design for achieving deterministic single-photon emission. (c) Thickness tuning at fixed geometrical parameters of $W_1 = 33$ nm, $W_2 = 39$ nm, $h = 84$ nm, $g = 162$ nm, $\alpha = 307$ nm, and $p = 410$ nm (See Figure 1 on the main manuscript for the position of these design parameters). The vertical dashed line indicates the Au thickness of 54 nm, at which the plasmon resonance matches the $MoSe_2$ exciton resonance. (d) Schematic of the $MoSe_2$ monolayer/alumina/Au metasurface/SiO_2 heterostructure. (e) Effect of the alumina (Al_2O_3) spacer on the polariton linewidth and (f) the corresponding equal-time second-order autocorrelation function, $g^{(2)}(0)$, under varying pure dephasing up to 40 meV. These results confirm that the designed hybrid structure can achieve high-purity single-photon emission.

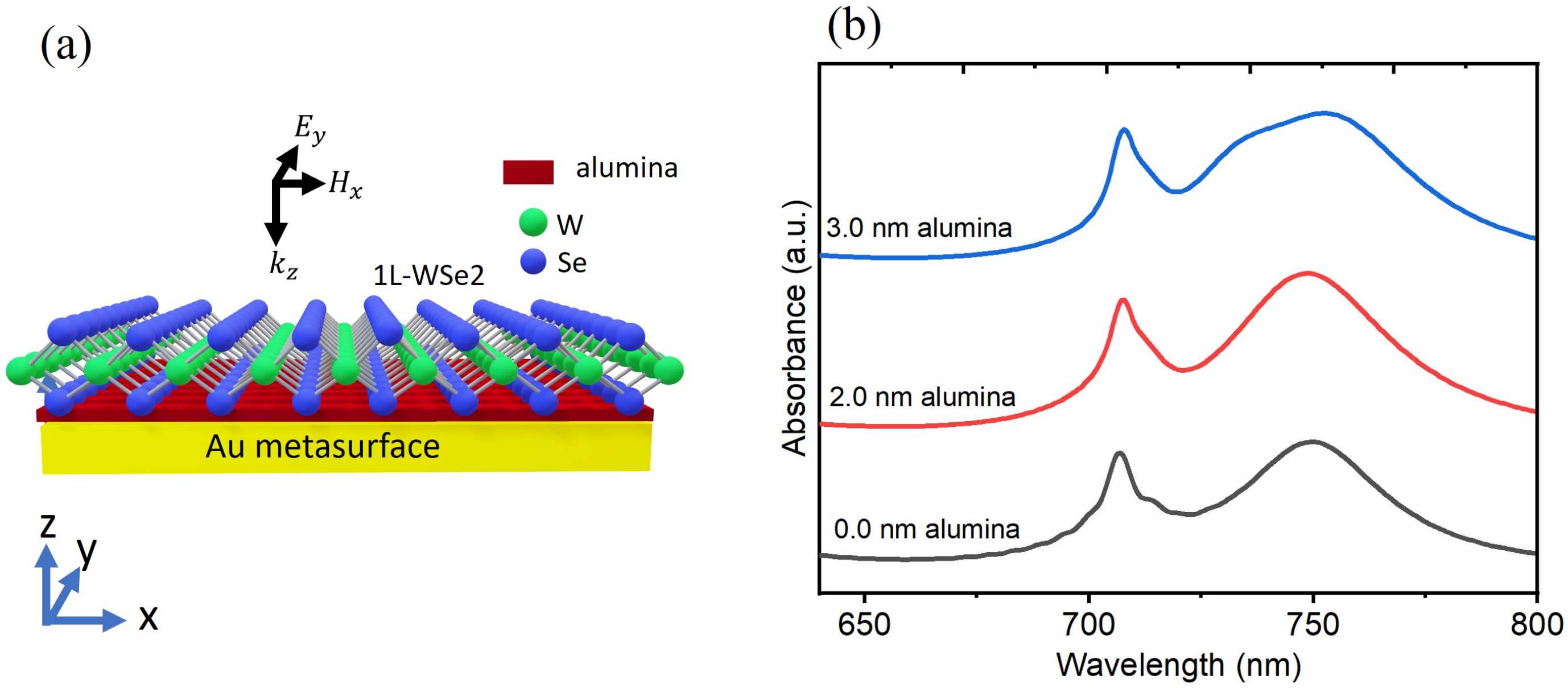

Figure S6: Effect of the alumina spacer thickness on the optical response of the Au–WSe_2 hybrid heterostructure. (a) The simulation was performed by inserting an alumina spacer layer between the WSe_2 monolayer and the Au metasurface, and (b) shows the corresponding polariton resonances with varying alumina thickness from 0 to 3 nm. It was confirmed that, although the lower polariton branch (LPB) broadens slightly with increasing alumina thickness, the strong coupling behavior is maintained. The design parmaters of the Au metasurfaces were optimised to $W_1 = 39$ nm, $W_2 = 33$ nm, $h = 157$ nm, $g = 108$ nm, $\alpha = 307$ nm, $t = 60$ nmand $p = 420$ nm.

References

[1] H. Zhong, L. Huang, S. Li, C. Zhou, S. You, L. Li, Y. Cheng, A. E. Miroshnichenko, *Applied Physics Reviews* **2024**, *11*, 3.

[2] C. Noh, *Scientific Reports* **2020**, *10*, 1 16076.
